# Efficacy and safety of eliapixant in endometriosis-associated pelvic pain: the randomized, placebo-controlled phase 2b SCHUMANN study

**DOI:** 10.1186/s12905-024-03188-8

**Published:** 2024-06-19

**Authors:** Susanne Parke, Kerstin Gude, Katrin Roth, Fabrizio Messina

**Affiliations:** 1 Research and Development, Bayer AG, Berlin, Germany; 2 Pharmacovigilance, Bayer AG, Berlin, Germany; 3 Data Science UK, Bayer PLC, UK

**Keywords:** Endometriosis-associated pelvic pain, Eliapixant, P2X3 receptor antagonist, Phase 2b clinical trial

## Abstract

**Background:**

The SCHUMANN study evaluated the efficacy and safety of the selective P2 × 3 antagonist eliapixant in patients with endometriosis-associated pelvic pain (EAPP).

**Methods:**

SCHUMANN was a randomized, placebo- and active comparator-controlled, double-blind to placebo and open-label to comparator, parallel-group, multicenter, dose-finding phase 2b study. The participants were women with surgically diagnosed endometriosis who fulfilled defined EAPP criteria. Participants were randomized 1:1:1:1 to twice daily (BID) 25 mg, 75 mg, or 150 mg oral eliapixant or a placebo for 12 weeks. An exploratory once-daily elagolix 150 mg treatment group was also included. The primary endpoint was the absolute change in mean worst EAPP from baseline to the end of intervention (EOI).

**Results:**

Overall, 215 participants were randomized for treatment (44 to eliapixant 25 mg, 44 to eliapixant 75 mg, 43 to eliapixant 150 mg, 43 to a placebo, and 41 to elagolix 150 mg). For safety reasons, the study was terminated early; both treatment and enrollment stopped immediately, producing less than 50% of the planned number of completers. The study found no significant differences in EAPP reduction from baseline between groups and no significant dose-response model. The elagolix 150 mg group showed better pain reduction than any of the other groups. No new safety signals were observed, relative to the previously known safety profile of eliapixant, which was generally well tolerated. However, one case of moderate and probably drug-induced liver injury in a participant receiving eliapixant 150 mg BID supported the association between eliapixant and a potential increase in liver function values, defined before the start of the phase 2 program.

**Conclusions:**

This study did not meet its primary objective as no statistically significant or clinically relevant differences in changes of mean worst EAPP from baseline were observed between treatment groups. The single observed case of moderate, probably drug-induced liver injury was the second case in the eliapixant phase 2 program conducted in the following indications: refractory or unexplained chronic cough, diabetic neuropathic pain, overactive bladder, and EAPP. Due to this, the benefit-risk ratio for the study was no longer considered to be positive.

**Clinical trial registration:**

ClinicalTrials.gov identifier NCT04614246; registered November 3, 2020.

**Supplementary Information:**

The online version contains supplementary material available at 10.1186/s12905-024-03188-8.

## Introduction

Endometriosis is a chronic, benign, sex-hormone-dependent inflammatory disease, characterized by the presence of endometrium-like tissue outside the uterine cavity [1–4] and causing a substantial burden to individuals and society [5–7]. Although its precise prevalence is unknown, estimates range from 1% to 15% within the general female population [5, 8–10]. Endometriosis is characterized by pain (primarily chronic, non-menstrual pelvic pain [NMPP]), pain during menstruation (dysmenorrhea) and sexual intercourse (dyspareunia), and infertility. The burden of the disease and the limitations of currently available treatment modalities have created a serious unmet medical need.

P2X3 is a non-selective cation channel activated by adenosine triphosphate (ATP); it has been described as a prominent mediator of pain [11]. P2X3 receptors are predominantly localized on primary sensory afferent neurons in small-to-medium diameter C and Aδ fibers throughout the body [11–13], which carry nerve impulses from sensory stimuli toward the central nervous system [12, 14]. Afferent neuronal hypersensitization via P2X3 receptor signaling plays a significant role in the pathways that trigger cough and bladder urgency and may influence the pathophysiology of endometriosis-associated chronic pain, overactive bladder (OAB), and diabetic neuropathic pain (DNP) [13, 15–19].

The expression of P2X3 receptors is upregulated during inflammation; this has been shown to sensitize peripheral nerves and contribute to central sensitization. Sensory nerve fibers within endometriotic lesions can themselves release inflammatory mediators (such as ATP, substance P, and nerve-growth factor) through a process known as neurogenic inflammation, which is thought to play a critical role in endometriosis-associated pain [20–24]. P2X3 expression in both endometriotic endometrium and lesions has been shown to be significantly higher than that found in control endometrium and positively correlated with pain. P2X3 channels are activated by ATP, which is released in response to various stimuli, including tissue injury, mechanical stress such as movement and distension, and the inflammation present in endometriotic lesions, particularly during menstruation [25–28]. Antagonizing P2X3 receptors was expected to combine high levels of pain relief with disease modification via an innovative mechanism.

Eliapixant is a potent P2X3 receptor antagonist that preferentially blocks the homomeric P2X3 channel. In a rat neurogenic inflammation model, eliapixant demonstrated robust efficacy in blocking inflammation of the skin evoked by an injection of mustard oil into the uterus and a concomitant transduction of the inflammation via the nervous system (i.e., neurogenic inflammation). It showed significant efficacy on visceral pain in a rat dyspareunia model and was still present 1 week after treatment, suggesting that eliapixant demonstrated efficacy beyond direct analgesic effects.

The goal of this project was to develop eliapixant as non-hormonal treatment for moderate-to-severe pain associated with endometriosis in women of reproductive age. Eliapixant was expected to provide clinical efficacy in the long-term management of pain associated with endometriosis, including a clinically meaningful reduction in chronic pelvic pain, dysmenorrhea, and dyspareunia, with a safety profile suitable for long-term use. It was also expected to improve patients’ quality of life substantially. At the start of this phase 2 study, these anticipated beneficial effects had not yet been demonstrated in patients with endometriosis-related pain. The phase 2b SCHUMANN study aimed to identify the optimal dose of eliapixant in patients with EAPP, to further assess efficacy, and to characterize the safety and tolerability profile of eliapixant over 12 weeks. Eliapixant has been developed in the indications OAB, refractory or unexplained chronic cough (RUCC), and DNP.

## Methods

### Study design

SCHUMANN (ClinicalTrials.gov NCT04614246) was a randomized, double-blind, open for active comparator, parallel-group, multicenter, phase 2b study to assess the efficacy and safety of three different doses of eliapixant (BAY 1817080) vs. placebo twice daily (BID) and elagolix 150 mg once daily in women with symptomatic endometriosis conducted at 144 centers in 20 countries across Europe, North America, China, and Japan (see Supplemental Methods for further details). The study comprised a 28-day screening phase, a 35-day pre-intervention period, an 84 + 3-day period of double-blind intervention, and a follow-up period lasting 38/90 days. When the protocol amendment was filed due to study termination, a comprehensive safety follow-up was implemented; this consisted of two additional visits for laboratory safety testing and a required 90-day follow-up for all participants, apart from those randomized to elagolix 150 mg (Supplemental Fig. S1). The latest version of the study protocol, including amendments and the statistical analysis plan, is available on ClinicalTrials.gov.

Eligible participants were centrally randomized 1:1:1:1:1 by the sponsor, using an interactive web response system (IWRS), to receive one of three oral doses of eliapixant BID (25 mg, 75 mg, or 150 mg; Bayer AG, Berlin, Germany), a placebo, or elagolix 150 mg once daily (QD). No participants from Japan or China were randomized to elagolix. To maintain blinding, tablets containing the placebo were identical in size, color, and shape to those containing eliapixant.

In addition to the study drugs described above, participants were only allowed to use standardized rescue medication (i.e., ibuprofen, acetaminophen, and tramadol) for treatment of endometriosis-associated pain. Women interested in participating in the study had to stop intake of hormonal treatments. Long-acting hormonal contraceptives as well as gonadotrophin-releasing hormone (GnRH) agonists, - GnRH antagonists, and progesterone receptor modulators had to be stopped at least 28 days before screening; all other hormonal therapies had to be stopped at Visit 1.

### Ethics approval and consent to participate

The Institutional Review Board/Independent Ethics Committee at each center approved the protocol. The study was carried out in accordance with Good Clinical Practice guidelines, the Declaration of Helsinki, and the International Ethical Guidelines of the Council for International Organizations of Medical Sciences. All participants provided written informed consent.

### Participants

The investigators enrolled women aged ≥ 18 years with endometriosis surgically diagnosed between 10 years and ≥ 8 weeks before screening and with self-reported moderate-to-severe pain. During the screening period, potential participants made at least 24 daily entries in an endometriosis symptom diary (ESD [29]), using the daily numeric rating scale (NRS) to indicate ESD item 1a (“worst pain”) summing up to ≥ 98. In Japan, inclusion by clinical diagnosis as opposed to surgical diagnoses was acceptable for up to 50% of the participants. Full inclusion and exclusion criteria are included in the Supplementary Methods.

### Procedures

Patient-reported outcome (PRO) assessments were collected using an electronic handheld diary (eDiary); pain catastrophizing and painDETECT scores were collected via paper questionnaires (see Supplemental Methods). Safety was monitored throughout the study (adverse events [AEs], clinical laboratory, and vital signs). Two questions on hair and eye color, previously associated with endometriosis and potentially useful in marking genetic subpopulations, were included among the baseline characteristics [30, 31].

### Study endpoints

The primary efficacy endpoint was the absolute change in mean worst EAPP (i.e., ESD item 1, measured daily via the NRS) from baseline to end of intervention (EOI). Other pre-specified endpoints included absolute change in mean worst EAPP from baseline to the first 4/8 weeks of intervention; absolute change in mean worst EAPP on bleeding and non-bleeding days from baseline to the first 4/8 weeks of intervention/EOI (measured via NRS by items 1 and 4 [i.e., intensity of vaginal bleeding, measured daily using a categorical response scale with five levels of increasing intensity: none, spotting, light, normal, heavy] of the ESD), item level, total and/or domain scores at screening, baseline, and Weeks 4/8/EOI and absolute change from baseline, as applicable, using the data collected by PROs (see Supplemental Methods) and absolute change in mean worst EAPP (overall, during, and outside days with vaginal bleeding) from EOI to non-overlapping, consecutive 28-day intervals during follow-up.

Treatment-emergent AEs (TEAEs) and serious AEs were recorded in line with the Medical Dictionary for Regulatory Activities (MedDRA), version 25.0. Additional safety assessments are described in the Supplemental Methods. At the end of the study, participants who spontaneously reported a taste-related AE completed an assessment on taste disturbances.

### Statistical analysis

A multiple comparison procedure modeling (MCP-Mod) approach [32] was used to complete a pre-specified analysis of the primary efficacy endpoint. As SCHUMANN was a phase 2b dose-finding study, the MCP-Mod approach, a well-accepted dose-finding method that uses available data more efficiently than traditional pairwise comparisons, was adopted [33, 34]. The MCP-Mod approach makes it possible to estimate a dose response and to select an optimum dose for further phase 3 trials [34].

To detect a dose–response signal, four candidate dose–response models were tested with a single contrast test using the generalized MCP-Mod approach. The null hypothesis (“the response to all doses is equal”) was tested against the alternative (“there is a dose–response relationship”). If at least one of the four individual model tests was statistically significant (adjusted *p* of one-sided test ≤ 0.1), a dose–response signal would be established. The model with the best fit would then be used to estimate the dose–response curve and minimum effective dose. For further information on the primary endpoint analysis, see the Supplementary Methods.

Sample size calculations were conducted to establish evidence of a drug effect across the doses. A sample size of 50 evaluable participants per dose group was predicted to have approximately 90% power to demonstrate a dose–response relationship for the primary efficacy endpoint, using a one-sided test at a type I error rate of α = 0.10 (see Supplementary Methods for more details).

The secondary endpoint analyses and definitions of the per-protocol as well as the full- and safety-analysis sets are described in the Supplementary Methods.

A statistical evaluation was performed using SAS software version 9.4 (SAS Institute, USA) and ValidR software version 3.5.2 or higher (Mango Solutions, UK). Confirmatory *p*-values are reported for the analysis of the primary endpoint. The study was not powered to make individual pairwise comparisons between dose groups. The analyses of secondary endpoints, sensitivity, and AEs should be viewed as exploratory.

## Results

**Fig. 1:**
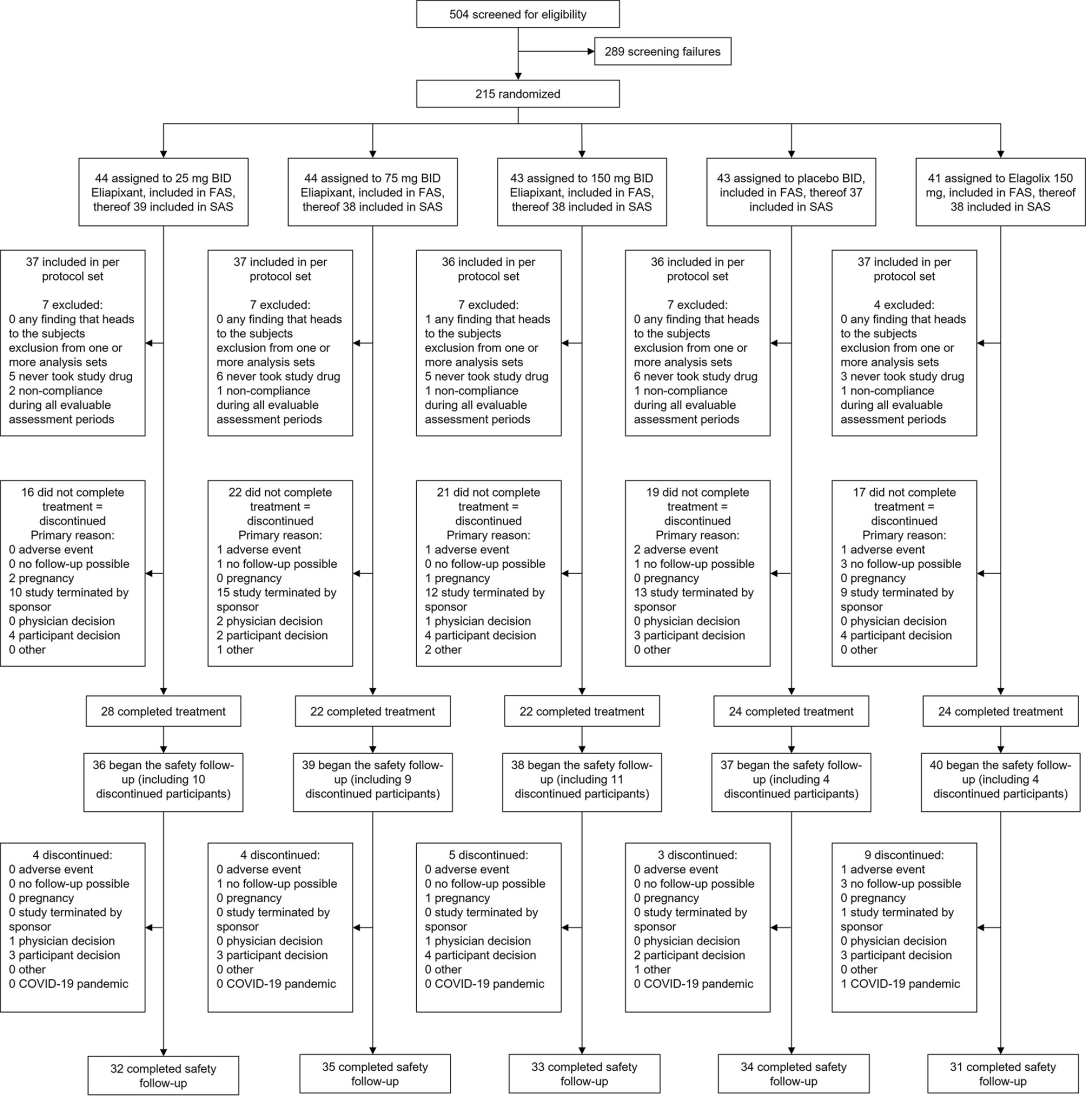
Participant disposition. *BID* twice daily, *FAS* full-analysis set, *SAS* safety-analysis set

In total, 504 participants were screened between 01/29/2021 and 01/27/2022, of whom 215 were randomized to eliapixant 25 mg (*n* = 44), 75 mg (*n* = 44), 150 mg (*n* = 43), placebo (*n* = 43) (all BID), or elagolix 150 mg (*n* = 41) (Fig. 1). Follow-up continued until 05/03/2022. Although all 215 randomized participants were included in the full-analysis set, 25 did not receive a study intervention; thus, the safety-analysis set includes 190 participants. A total of 183 participants were included in the per-protocol set (for a definition see Supplemental Methods). In total, 120 participants (42.7%) completed the treatment period.

Table 1 presents an overview of per-protocol set demographic characteristics. Similar results were obtained in the full-analysis set. Age and body mass index (BMI) were generally well balanced across the treatment arms (Table 1). The mean (standard deviation [SD]) age was 34.64 (7.22) years, with 26.8% of participants aged < 30 years, 50.3% aged 30–40 years, and 23.0% aged > 40–55 years. The mean (SD) BMI was 25.54 (5.89) kg/m^2^. In the per-protocol set, 73.8% of participants were white. In the elagolix 150 mg arm, however, 94.6% were white, as participants from Japan and China could not be randomized to elagolix (Table 1).

**Table 1:** Selected demographics and clinical characteristics (per-protocol set)

	Eliapixant 25 mg BID *n* = 37 (100%)	Eliapixant 75 mg BID *n* = 37 (100%)	Eliapixant 150 mg BID *n* = 36 (100%)	Placebo *n* = 36 (100%)	Elagolix 150 mg *n* = 37 (100%)	Total *n* = 183 (100%)
Race						
White	26 (70.3%)	26 (70.3%)	25 (69.4%)	23 (63.9%)	35 (94.6%)	135 (73.8%)
Black or African American	0	1 (2.7%)	2 (5.6%)	2 (5.6%)	0	5 (2.7%)
Asian	11 (29.7%)	10 (27.0%)	9 (25.0%)	11 (30.6%)	0	41 (22.4%)
Not reported	0	0	0	0	2 (5.4%)	2 (1.1%)
Age (years)	33.41 (7.07)	36.32 (7.60)	35.75 (7.11)	35.58 (7.17)	32.19 (6.64)	34.64 (7.22)
Age group						
< 30 years	11 (29.7%)	7 (18.9%)	7 (19.4%)	10 (27.8%)	14 (37.8%)	49 (26.8%)
30–40 years	20 (54.1%)	18 (48.6%)	21 (58.3%)	14 (38.9%)	19 (51.4%)	92 (50.3%)
> 40–55 years	6 (16.2%)	12 (32.4%)	8 (22.2%)	12 (33.3%)	4 (10.8%)	42 (23.0%)
Main eye color						
Green	4 (10.8%)	8 (21.6%)	7 (19.4%)	8 (22.2%)	12 (32.4%)	39 (21.3%)
Blue	8 (21.6%)	7 (18.9%)	6 (16.7%)	5 (13.9%)	10 (27.0%)	36 (19.7%)
Gray	1 (2.7%)	2 (5.4%)	3 (8.3%)	1 (2.8%)	2 (5.4%)	9 (4.9%)
Hazel	1 (2.7%)	1 (2.7%)	4 (11.1%)	4 (11.1%)	0	10 (5.5%)
Brown	23 (62.2%)	19 (51.4%)	16 (44.4%)	18 (50.0%)	13 (35.1%)	89 (48.6%)
Natural hair color (at age 18)						
Red	0	1 (2.7%)	1 (2.8%)	0	0	2 (1.1%)
Blonde	7 (18.9%)	4 (10.8%)	8 (22.2%)	7 (19.4%)	8 (21.6%)	34 (18.6%)
Light brown	9 (24.3%)	10 (27.0%)	8 (22.2%)	8 (22.2%)	14 (37.8%)	49 (26.8%)
Dark brown	10 (27.0%)	13 (35.1%)	7 (19.4%)	6 (16.7%)	14 (37.8%)	50 (27.3%)
Black	11 (29.7%)	9 (24.3%)	12 (33.3%)	15 (41.7%)	1 (2.7%)	48 (26.2%)
Body mass index (kg/m^2^)	25.08 (6.53)	26.01 (5.17)	25.34 (5.24)	25.61 (6.18)	25.64 (6.44)	25.53 (5.89)
Mean worst pain	6.09 (1.73)	6.05 (1.78)	6.63 (1.62)	5.89 (1.93)	6.38 (1.78)	6.21 (1.77)
Non-menstrual pelvic pain	5.85 (1.81)	5.80 (1.95)	6.45 (1.78)	5.62 (2.05)	6.18 (1.86)	5.98 (1.89)
Dysmenorrhea	7.16 (1.60)	6.91 (1.55)	7.38 (1.39)	7.00 (1.77)	7.29 (1.84)	7.15 (1.63)

Abbreviations: *BID* twice daily, *SD* standard deviation

Data are expressed as mean (SD) unless otherwise stated

Baseline demographics and clinical characteristics were generally well balanced across the treatment groups (Table 1). Slight imbalances between the treatment groups were observed in relation to mean worst pain, NMPP, and dysmenorrhea. The mean (SD) of mean worst pain, NMPP, and dysmenorrhea was higher in the eliapixant 150 mg BID arm (6.63 [1.62], 6.45 [1.78], and 7.38 [1.39]), respectively, and in the elagolix 150 mg arm (6.38 [1.78], 6.18 [1.86], and 7.29 [1.84]), respectively, than in other arms (Table 1).

Slight imbalances between the treatment groups were also observed with regard to hair and eye color. As expected, given the participating countries, about half of the population (48.6%) had brown eyes. However, the proportion ranged between 35.1% in the elagolix 150 mg arm, which had predominantly white participants (no women from China or Japan were randomized to this treatment group), and 62.2% in the eliapixant 25 mg group. The full-analysis set results were similar.

The data for the primary efficacy endpoint, absolute change in mean worst EAPP from baseline to EOI (measured daily on the NRS as ESD item 1) are shown in Table 2.

**Table 2:** Primary endpoint: mean worst EAPP and absolute change from baseline to end of intervention (measured daily on the NRS as ESD item 1)—primary per-protocol set

Timepoint		Eliapixant 25 mg BID	Eliapixant 75 mg BID	Eliapixant 150 mg BID	Placebo	Total
Baseline	*n*	31	31	31	30	123
	Missing	0	0	0	0	0
	Mean (SD)	6.40 (1.66)	6.13 (1.77)	6.95 (1.38)	6.14 (1.96)	6.41 (1.72)
	Median	6.68	6.25	6.96	5.74	6.62
	Q1, Q3	5.27, 7.44	4.86, 7.46	6.19, 7.86	4.56, 7.64	5.12, 7.58
	Min, max	3.1, 9.6	3.0, 9.6	3.8, 9.9	2.6, 10.0	2.6, 10.0
Week 12 (EOI)	*n*	24	22	20	26	92
	Missing	7	9	11	4	31
	Mean (SD)	4.57 (2.10)	4.15 (2.49)	5.15 (2.42)	4.29 (1.71)	4.52 (2.17)
	Median	4.61	3.91	4.86	3.95	4.18
	Q1, Q3	3.15, 5.99	1.61, 6.54	3.30, 7.13	3.08, 5.80	3.09, 6.13
	Min, max	0.5, 8.5	0.6, 8.6	0.5, 10.0	1.5, 8.3	0.5, 10.0
Change from baseline	*n*	24	22	20	26	92
	Missing	7	9	11	4	31
	Mean (SD)	–1.56 (1.35)	–2.12 (2.66)	–1.88 (2.03)	–1.89 (1.91)	–1.86 (2.00)
	Median	–1.36	–1.39	–1.30	–1.67	–1.40
	Q1, Q3	–2.13, − 0.50	–3.29, − 0.17	–3.46, − 0.19	–3.04, − 0.39	–3.01, − 0.35
	Min, max	–4.1, 0.4	–7.9, 1.9	–6.2, 1.6	–6.3, 1.3	–7.9, 1.9

Abbreviations: *BID* twice daily, *EAPP* endometriosis-associated pelvic pain (item 1 in the Endometriosis Symptom Diary [*ESD*]), *EOI* end of intervention, *NRS* numeric rating scale, *Q1* first quartile, *Q3* third quartile, *SD* standard deviation

At Week 12, the end of the intervention, the mean (SD) changes in mean worst EAPP from baseline were − 1.56 (1.35) in the eliapixant 25 mg BID arm, − 2.12 (2.66) in the eliapixant 75 mg BID arm, − 1.88 (2.03) in the eliapixant 150 mg BID arm, and − 1.89 (1.91) in the placebo arm, based on the primary per-protocol set. The full-analysis set results were similar. For elagolix, the open-label comparator arm, the mean (SD) of mean worst EAPP was 6.38 (1.76) at baseline and 3.39 (2.57) at Week 12 (EOI), resulting in a mean (SD) change from baseline of − 2.83 (2.38), based on the full-analysis set.

The least squares (LS) mean (standard error [SE]) of change obtained via mixed-model repeated measures from baseline to Week 12 (EOI) was − 1.63 (0.38) in the eliapixant 25 mg BID arm, − 2.13 (0.41) in the eliapixant 75 mg BID arm, − 1.96 (0.41) in the eliapixant 150 mg BID arm, and − 1.94 (0.38) in the placebo arm. Overall, no significant differences were observed across the various treatment arms.

**Fig. 2:**
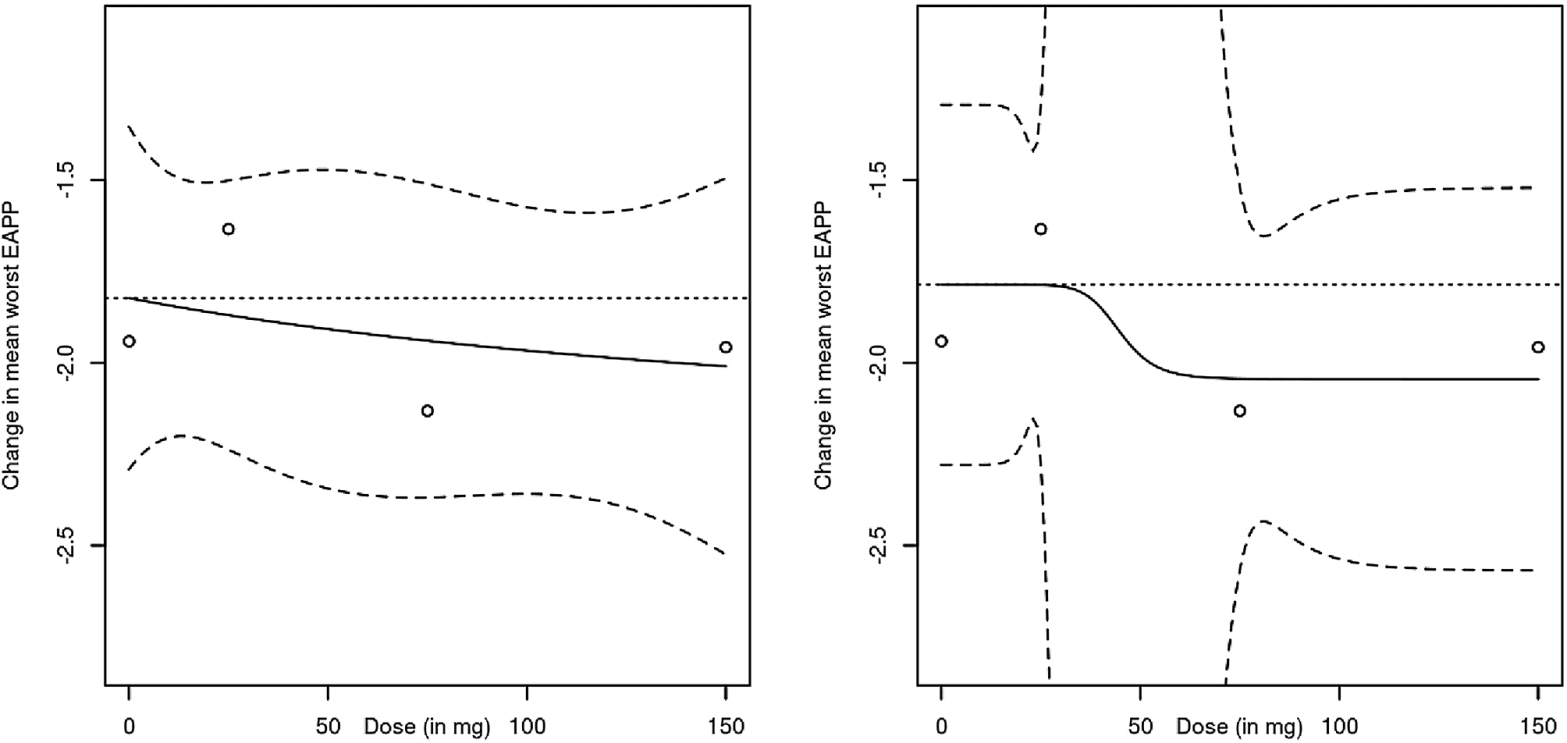
The dose response model and target dose for the change over 28 days indicate the change in worst EAPP from baseline to Week 12 (with 80% CI). Left: Emax model; right: SigEmax model. *CI* confidence interval, *EAPP* endometriosis-associated pelvic pain

At Week 12 (EOI) the adjusted *p*-values for the Emax 1, Emax 2, SigEmax 1, and SigEmax 2 candidate models were 0.5266, 0.4679, 0.4052, and 0.4082, respectively. No significant dose-response model was found (Fig. 2).

A secondary analysis on the primary per-protocol set, including the elagolix arm, resulted in similar LS mean changes from baseline for the placebo and eliapixant arms, but showed an LS mean change (SD) from baseline of − 2.69 (0.41) for the elagolix arm, resulting in a difference of approximately − 0.8 from the placebo. This difference is comparable to the effect of elagolix 150 mg QD observed in previous studies [35]. The mean EAPP on bleeding days was higher at baseline than the overall EAPP, with a mean (SD) of 7.19 (1.62) across all treatment groups and a change from baseline ranging from − 1.07 (1.7) to − 1.73 (2.1), a smaller effect than those observed for overall EAPP. Mean EAPP on non-bleeding days was slightly lower at baseline than overall EAPP, with a mean (SD) of 6.00 (1.87) across all treatment groups and a change from baseline similar to that observed for overall EAPP. No relevant dose–response relationship was observed for any of these endpoints.

Throughout the entire study, participants were asked to report pain at its worst during the past 24 h on a 0–10 NRS on the eDiary to assess dyschezia and dysuria. At baseline, dyschezia mean worst pain was present across all treatment groups with a mean (SD) of 3.78 (2.89), and the means (SDs) in the different treatment groups ranging from 3.04 (2.83) in the eliapixant 25 mg BID group to 4.15 (2.68) in the elagolix 150 mg group. After 12 weeks of treatment, a reduction was observed in all treatment groups, with mean (SD) changes from baseline of − 0.65 (0.81) in the eliapixant 25 mg BID group up to − 2.18 (1.95) in the elagolix 150 mg group. Dysuria mean worst pain at baseline was observed with an overall mean (SD) of 2.80 (2.79), ranging from 2.37 (2.82) in the eliapixant 25 mg BID group to 3.40 (2.78) in the elagolix 150 mg group. After 12 weeks of treatment, a reduction was observed in all treatment groups, with mean (SD) changes from baseline of − 0.67 (1.11) in the eliapixant 25 mg BID group up to − 1.67 (2.01) in the elagolix 150 mg group. No dose-response trend and no relevant difference to placebo were observed for dyschezia or dysuria in the different eliapixant groups.

A higher percentage of participants experienced TEAEs in the eliapixant 150 mg BID arm (76.3% [*n* = 29]), placebo arm (73.0% [*n* = 27]), and elagolix 150 mg arm (73.7% [*n* = 28]) than did so in the other two lower-dose eliapixant treatment arms (eliapixant 25 mg BID (59.0% [*n* = 23]) and eliapixant 75 mg BID (55.3% [*n* = 21]). The same pattern was seen in the percentage of participants with TEAEs attributed to study intervention (Table 3). No AEs/TEAEs had death as an outcome. Few participants had serious TEAEs (Table 3). Two serious TEAEs were assessed as related to the study intervention: one in the eliapixant 150 mg BID arm (MedDRA preferred term [PT] “Liver function test increased”) and one in the elagolix 150 mg arm (MedDRA PT “Hypertensive crisis”). A low percentage of participants stopped using the study drug permanently due to a TEAE (Table 3).

**Table 3:** Adverse events: overall summary (safety-analysis set)

	Eliapixant 25 mg BID *n* = 39 (100%)	Eliapixant 75 mg BID *n* = 38 (100%)	Eliapixant 150 mg BID *n* = 38 (100%)	Placebo *n* = 37 (100%)	Elagolix 150 mg QD *n* = 38 (100%)
Any AE	33 (84.6%)	28 (73.7%)	32 (84.2%)	30 (81.1%)	32 (84.2%)
Any SAE	1 (2.6%)	2 (5.3%)	3 (7.9%)	1 (2.7%)	2 (5.3%)
Any AE resulting in death	0	0	0	0	0
Any AE resulting in permanent discontinuation of the study drug	0	1 (2.6%)	1 (2.6%)	2 (5.4%)	1 (2.6%)
Any TEAE	23 (59.0%)	21 (55.3%)	29 (76.3%)	27 (73.0%)	28 (73.7%)
Any drug-related TEAE	4 (10.3%)	6 (15.8%)	10 (26.3%)	10 (27.0%)	13 (34.2%)
Any serious TEAE	0	2 (5.3%)	2 (5.3%)	1 (2.7%)	1 (2.6%)
Any drug-related serious TEAE	0	0	1 (2.6%)	0	1 (2.6%)
Any TEAE resulting in death	0	0	0	0	0
Any TEAE resulting in permanent discontinuation of the study drug	0	1 (2.6%)	1 (2.6%)	2 (5.4%)	1 (2.6%)

Abbreviations: *AE* adverse event, *BID* twice daily, *QD* once daily, *SAE* serious adverse event, *TEAE* treatment-emergent adverse event

TEAEs were reported from the start of study intervention to 14 days after the last study medication intake

Table 4 summarizes the most commonly reported TEAEs in each treatment arm, with headache being the most frequently reported TEAE across all treatment arms. In almost all safety-analysis set participants, the maximum intensity of TEAEs was assessed as either mild (43.7%) or moderate (20.0%). Only 7 participants reported severe TEAEs: 3 participants in the eliapixant 150 mg arm, 2 participants in the placebo arm, and 2 participants in the elagolix 150 mg arm.

**Table 4:** TEAEs: Two most frequent PTs in each treatment group–number (%) of participants (safety-analysis set)

PT MedDRA version 25.0	Eliapixant 25 mg BID *n* = 39 (100%)	Eliapixant 75 mg BID *n* = 38 (100%)	Eliapixant 150 mg BID *n* = 38 (100%)	Placebo *n* = 37 (100%)	Elagolix 150 mg QD *n* = 38 (100%)
Headache	**7 (17.9%)**	**7 (18.4%)**	**6 (15.8%)**	**9 (24.3%)**	**8 (21.1%)**
Activated partial thromboplastin time prolonged	**3 (7.7%)**	2 (5.3%)	2 (5.3%)	**4 (10.8%)**	5 (13.2%)
Blood fibrinogen decreased	**3 (7.7%)**	0	0	1 (2.7%)	3 (7.9%)
COVID-19	**3 (7.7%)**	2 (5.3%)	0	0	1 (2.6%)
Pyrexia	**3 (7.7%)**	1 (2.6%)	2 (5.3%)	3 (8.1%)	0
Nasopharyngitis	1 (2.6%)	**4 (10.5%)**	1 (2.6%)	2 (5.4%)	2 (5.3%)
Hot flush	0	0	0	2 (5.4%)	**7 (18.4%)**
Nausea	0	1 (2.6%)	**5 (13.2%)**	1 (2.7%)	2 (5.3%)
Vaccination-site pain	0	**4 (10.5%)**	0	4 (10.8%)	0

Abbreviations: *AE* adverse event, *BID* twice daily, *MedDRA* Medical Dictionary for Regulatory Activities, *PT* preferred term, *QD* once daily, *SOC* System Organ Class, *TEAE* treatment-emergent adverse event

AEs were sorted by descending frequency of PTs of the MedDRA classification in the eliapixant 25 mg BID arm. A participant was counted only once within each primary SOC and preferred term

Most TEAEs attributed to study intervention affected single participants and were assessed as having either mild or moderate maximum intensity. The TEAEs attributed to study intervention and reported by at least 2 participants were as follows:

- In the eliapixant 75 mg BID arm: 2 participants reported intermenstrual bleeding.
- In the eliapixant 150 mg BID arm: 2 participants reported dysgeusia, 2 participants reported taste disorder, and 3 participants reported hypogeusia.
- In the elagolix 150 mg arm: 2 participants reported headache and 6 participants reported hot flushes.

For 1 participant in the placebo arm (PT: “Nightmares”) and 2 participants in the elagolix 150 mg arm (PT: “Hot flushes”, PT: “Hypertensive crisis”), TEAEs attributed to study intervention were assessed as severe at maximum intensity.

Ten taste-related AEs were reported in the eliapixant 150 mg BID (6 participants, 15.8%; one of those reported three events) and placebo arms (2 participants, 5.4%). Eight of the 10 taste-related AEs were attributed to the study medication and impacted participants in the eliapixant 150 mg arm. No participant discontinued treatment due to a taste-related AE.

Increases in liver function parameters and antithrombin III activity were described as potential risks in the study protocol and monitored closely across the study period. No mean increase over time or relevant differences were observed across the treatment arms in relation to the mean values of aspartate aminotransferase (AST), alanine aminotransferase (ALT), gamma-glutamyl transferase (GGT), or bilirubin. However, a dose-dependent increase in the mean values of alkaline phosphatase (AP) was seen at Week 2 after the start of the study intervention. From Week 4 onward, the values remained relatively stable until the end of intervention at Week 12. All changes were reversible and returned to baseline levels by the safety follow-up visit—approximately 38 days after the end of the intervention. The clinical relevance of this finding and the origins of the increase (i.e., liver vs. bone) are unclear in the absence of a concurrent increase in the mean ALT, AST, bilirubin, or GGT values. In total, 4 participants (10.3%) in the eliapixant 25 mg BID arm, 3 participants (7.9%) in the eliapixant 75 mg BID arm, 1 participant (2.6%) in the eliapixant 150 mg BID arm, 2 participants (5.4%) in the placebo arm, and 3 participants in the elagolix 150 mg arm reported TEAEs associated with the Standardized MedDRA Query “Drug-related hepatic disorders”. One participant in the eliapixant 150 mg BID arm experienced a drug-induced liver injury, reported as a suspected unexpected serious adverse reaction (SUSAR). The 44-year-old study participant was diagnosed with “liver function test increased” (AST 4.8-fold upper limit of normal [ULN], ALT 7.5-fold ULN) at her regular visit after 4 weeks of treatment with the study drug; normal values had been recorded at screening and baseline and slightly increased values at Week 2 after the start of treatment. Unblinding revealed that the patient had received a dose of 150 mg BID eliapixant. Other liver parameters remained within normal ranges. Treatment with the study drug was discontinued immediately. This patient reported malaise and edema starting 3 weeks after the treatment began. She recovered from her symptoms and her transaminases returned to normal 2 weeks (AST) and 4 weeks (ALT) after the treatment ended. A detailed analysis of alternative causes of this patient’s moderate liver injury, based on the Food and Drug Administration 2009 Drug-Induced Liver Injury Guideline [36], found that she was reactive for anti-hepatitis A virus (HAV) immunoglobulin M (IgM) antibodies at baseline and at 2, 4, and 6 weeks thereafter as a potential alternative cause. However, no HAV ribonucleic acid or anti-HAV immunoglobulin G (IgG) antibodies were detected in serum at any point in time (unfortunately, no stool sample was collected or analyzed). Furthermore, no anti-HAV IgG antibodies were detected until 10 weeks after the first anti-HAV IgM antibodies were found; earlier detection would be expected during the course of a typical HAV infection. The totality of data gathered to clarify the causality of this case did not support a recent acute infection by HAV. In conclusion, the case was assessed by an external hepatology expert as an acute hepatocellular liver injury of moderate severity, based on the associated symptoms. The suggestive chronology and the absence of any other causes with clear potential made eliapixant the most likely cause of the liver injury.

A mean and median increase in antithrombin activity was observed in the eliapixant arms but not in the placebo arm, in line with similar findings from other phase 1 and 2 studies with eliapixant [37–39]. However, no differences were observed in TEAEs involving bleeding between the treatment arms.

A mean increase in fibrinogen was seen in the eliapixant arms, but not in the placebo arm, confirming a similar finding in other phase 2 studies with eliapixant [38, 39]. The clinical relevance remains unclear, as there was no difference between eliapixant and the placebo regarding potential clinical manifestations of such changes, e.g., frequency of TEAEs involving bleeding or thromboembolic events.

Six participants reported a pregnancy during this study: 2 in the placebo arm and 1 in the elagolix 150 mg arm. Three participants reported pregnancies conceived during treatment: 2 in the eliapixant 25 mg arm and 1 in the eliapixant 150 mg arm.

## Discussion

Endometriosis, a debilitating gynecologic condition affecting millions of women worldwide, poses significant challenges in both diagnosis and management. A general lack of awareness by women and healthcare providers results in a significant delay from when a woman first experiences symptoms until she eventually is diagnosed and treated. Despite its prevalence and profound impact on quality of life, there remains a critical need for further research and the development of more effective treatment options [40].

To date, none of the medical treatments have been able to cure the disease, most treatments are not suitable for long-term use due to side effects [41], and symptoms recur as soon as the medication is stopped. Hormone treatments for endometriosis include combined contraceptives, progestogens, GnRH agonists, GnRH antagonists, and aromatase inhibitors. All treatments lead to a clinically significant reduction in pain with a similar magnitude of the treatment effect. Symptoms return after cessation of treatment and all hormones used to manage endometriosis have unwanted side effects [42]. Non-steroidal anti-inflammatory drugs are effective in reducing EAPP, but also have significant side effects, including gastric ulceration. Surgery can be effective to remove endometriosis lesions and scar tissue, but success rates are dependent on the extent of disease and the surgeon’s skills.

The SCHUMANN study aimed to assess the efficacy and safety of three different doses of eliapixant, in comparison with a placebo and elagolix 150 mg, in women with symptomatic endometriosis. The study was terminated prematurely, due to liver safety concerns; as a result, less than 50% of the planned number of participants completed the study.

Reductions in mean worst EAPP were observed at Week 12 in all treatment groups; no significant differences were observed across the treatment arms or compared with a placebo. At end of the intervention (Week 12), the mean (SD) changes in mean worst EAPP from baseline were − 1.56 (1.35) in the eliapixant 25 mg BID arm, − 2.21 (2.66) in the eliapixant 75 mg BID arm, − 1.88 (2.03) in the eliapixant 150 mg BID arm, and − 1.89 (1.91) in the placebo arm (primary per-protocol set). Only the elagolix 150 mg arm achieved better pain reduction than the other treatment arms of − 2.83 (2.38) (full-analysis set). The observed efficacy of elagolix was in line with expectations.

No significant dose-response model was found at any time. As no statistically significant or clinically relevant differences were observed between the treatment groups, this study did not meet its primary objective.

Due to the low percentage of women who completed the study, it was not considered meaningful to carry out subgroup analyses, for example with regard to hair and eye color.

No new safety signals were observed in relation to the known safety profile of eliapixant, which was generally well tolerated in this study. More participants experienced TEAEs in the eliapixant 150 mg BID, placebo, and elagolix 150 mg arms than in the two lower-dosed eliapixant treatment arms; no specific event caused this difference between the treatment arms.

Increases were observed in mean antithrombin activity, fibrinogen, and AP, confirming similar findings from other phase 2 studies with eliapixant [37–39]. The clinical relevance of these findings remains unclear, as there was no difference between eliapixant and the placebo with regard to clinical manifestations of these changes (e.g., TEAEs related to bleeding or thromboembolic events). Taste-related AEs were observed only in the eliapixant 150 mg BID (6 participants, 15.8%) and placebo (2 participants, 5.4%) arms.

The one observed case of moderate, probably drug-induced liver injury in a SCHUMANN participant receiving eliapixant 150 mg BID was the second case in the eliapixant phase 2 program with the following indications: RUCC, DNP, OAB, and EAPP. The first case occurred in a 26-year-old female participant after 4 weeks of exposure to eliapixant 150 mg BID in the phase 2b study of patients suffering from RUCC [38]. Although increases in liver function parameters with eliapixant treatment had been defined as a potential risk before the start of phase 2 studies, the benefit-risk ratio for the SCHUMANN study was no longer considered positive following two cases of moderate drug-induced liver injury of hepatocellular origin in participants exposed to eliapixant for 8–12 weeks of treatment during the phase 2 program in all indications and considering the totality of liver-safety data from the phase 2 program. The study was therefore put on clinical hold with an immediate stop of treatment and enrollment.

The strengths of SCHUMANN include its baseline demographics, which largely reflect those seen in a typical endometriosis population. The recruitment of participants across 20 countries meant that the results were likely to reflect the global population of patients with endometriosis. The limitations of the study include its premature termination, which resulted in less than 50% of the planned number of completers.

In summary, the SCHUMANN study did not show any significant differences in mean worst EAPP reductions at Week 12 across various treatment arms or compared with a placebo. The tolerability profile of eliapixant was consistent with that observed in other phase 2 studies of the program. However, the benefit-risk ratio for this study was no longer considered positive following the second case of a moderate, probably drug-induced liver injury in a participant receiving eliapixant 150 mg BID in the phase 2 program. The study was terminated prematurely; subsequently, Bayer AG discontinued the entire development program in all indications.

Overall, there is need for further research in the fields of new treatment options and early diagnosis of endometriosis, particularly in young patients.

## Availability of data and materials

The availability of data underpinning this publication will be determined in accordance with Bayer’s commitment to the European Federation of Pharmaceutical Industries and Associations and Pharmaceutical Research and Manufacturers of America and their principles for responsible clinical trial data sharing, pertaining to scope, timepoint, and the data access process. At the request of qualified scientific and medical researchers, Bayer commits to sharing patient-level clinical trial data, study-level clinical trial data, and protocols from patient clinical trials of medicines and indications approved in the US and European Union, as required for legitimate research. This commitment applies to data on new medicines and indications approved by the European Union and US regulatory agencies on or after January 1, 2014. Interested researchers can use www.clinicalstudydatarequest.com to request access to anonymized patient-level data and supporting documents from clinical studies in order to carry out further research to advance medical science or improve patient care. Alternatively, researchers may contact Susanne Parke (Research and Development, Bayer AG, Berlin, Germany; susanne.parke@bayer.com). Information on the Bayer criteria for listing studies and other relevant information is provided in the study sponsors section of the portal. Access will be granted to anonymized patient-level data, protocols, and clinical study reports following approval by an independent scientific review panel. Bayer is not involved in the decisions made by the independent review panel and will implement all necessary measures to safeguard patient privacy.

### Electronic supplementary material

Below is the link to the electronic supplementary material.

Supplementary Material 1

## Data Availability

No datasets were generated or analyzed during the current study.

## Abbreviations

AE: Adverse event
ALT: Alanine aminotransferase
AP: Alkaline phosphatase
AST: Aspartate aminotransferase
ATP: Adenosine triphosphate
BID: Twice daily
BMI: Body mass index
CI: Confidence interval
DNP: Diabetic neuropathic pain
EAPP: Endometriosis-associated pelvic pain
eDiary: Electronic handheld diary
EOI: End of intervention
ESD: Endometriosis symptom diary
GGT: Gamma-glutamyl transferase
GnRH: Gonadotrophin-releasing hormone
HAV: Anti-hepatitis A virus
IgG: Immunoglobulin G
IgM: Immunoglobulin M
LS: Least squares
MCP-Mod: Multiple comparison procedure modeling
MedDRA: Medical Dictionary for Regulatory Activities
NMPP: Non-menstrual pelvic pain
NRS: Numeric rating scale
OAB: Overactive bladder
PRO: Patient-reported outcome
PT: Preferred term
QD: Once daily
RUCC: Refractory or unexplained chronic cough
SAE: Serious adverse event
SD: Standard deviation
TEAE: Treatment-emergent adverse event
ULN: Upper limit of normal
