# Supplementary material for: Efficacy and safety of eliapixant in endometriosis-associated pelvic pain: the randomized, placebo-controlled phase 2b SCHUMANN study
Source: BMC Womens Health. 2024 Jun 19;24:353. doi: 10.1186/s12905-024-03188-8 (PMC11186168; doi:10.1186/s12905-024-03188-8)
Supplement: Supplementary file 1 — Supplementary Material 1 [file 12905_2024_3188_MOESM1_ESM.docx]

# Supplemental Material

# Efficacy and safety of eliapixant in endometriosis-associated pelvic pain: the randomized, placebo-controlled phase 2b SCHUMANN study

Susanne Parke^1*^, Kerstin Gude^2^, Katrin Roth^1^ and Fabrizio Messina^3^

^1^ Research and Development, Bayer AG, Berlin, Germany

^2^ Pharmacovigilance, Bayer AG, Berlin, Germany

^3^ Data Science UK, Bayer PLC, UK

The full list of investigators is provided in the Supplemental Material

Corresponding author:

Susanne Parke
susanne.parke@bayer.com

# Contents

## Supplementary 1: SCHUMANN Study Investigators

## Supplementary 2: Supplemental Methods

Participating regions and countries

Full inclusion criteria

Full exclusion criteria

Additional information on procedures

Additional information on safety-related assessments

Additional information on the statistical analysis

## Supplementary 3: Supplemental Results

EAPP at 4/8 weeks

Rescue medication

EIS (domain scores)

PainDETECT

Additional results based on PROs

## Supplementary 4: Supplemental Tables and Figures

## Supplementary 5: Supplemental References

## SCHUMANN Study Investigators

**AUSTRIA**Prof. Rene Wenzl

Prof. Hans-Christian Egarter

Prof. Johannes Ott

Dr. Gerda Trutnovsky

**BELGIUM**

Dr. Wim Decleer

Prof. Jean-Luc Squifflet

Dr. Peter Sieprath

Dr. Steven Weyers

**BULGARIA**

Dr. Milka Saldzhiyska-Veleva

Dr. Dimka Zantina

Dr. Nikolay Genov

Dr. Georgi Borikov

Dr. Elis Ismail

**CANADA**

Dr. Sukhbir Singh

Dr. Francois Bissonnette

Dr. Maya Naccache

**CHINA**

Prof. Yingfang Zhou

Dr. Ping Su

Wei Wang

Dr. Yaojuan He

Prof. Xinmei ZHANG

Dr. Xiaoyan Ying

Prof. Yudi Zhang

**CZECH REPUBLIC**

Dr. Robert Hudecek

Dr. Leopold Rotter

Dr. Peter Koliba

Dr. Tereza Smrhova-Kovacs

Dr. Michael Svec

Dr. Ondrej Mika

Dr. Hana Kosova

Dr. Olga Hlavackova

Dr. Martin Charvat

**ESTONIA**

Dr. Deniss Soritsa

Dr. Kadri-Liina Vahula

**FINLAND**

Prof. Oskari Heikinheimo

Dr. Jatta Vallirinne

**GERMANY**

Dr. Andrea Heweker

Prof. Martin Sillem

Prof. Sylvia Mechsner

Dr. Saskia Kerschischnik

Prof. Ariane Germeyer

**GREECE**

Prof. Nikolaos Vlahos

Prof. Georgios Adonakis

**HUNGARY**

Dr. Gabor Nemeth

Dr. Arpad Rucz

**ITALY**

Prof. Valentino Bergamini

Prof. Paolo Vercellini

Prof. Felice Petraglia

Prof. Angelo Cagnacci

Prof. Giovanni Scambia

Dr. Antonio Simone Laganà

**JAPAN**

Dr. Miho Fujii

Dr. Shinichiro Wada

Dr. Akira Nishikawa

Dr. Toshiro Mizutani

Dr. Masato Sakai

Dr. Masatoshi Sugita

Dr. Haruya Saji

Dr. Yuji Kashiwazaki

Dr. Akinori Oki

Dr. Hajime Tsunoda

Dr. Takashi Nakano

Dr. Mamoru Urabe

Dr. Fuminori Kitada

Dr. Mari Sawada

Dr. Kei Hirabayashi

Dr. Kiyonori Miura

Dr. Akira Fujishita

Dr. Ken Sugiura

Dr. Futoshi Arakane

Dr. Eiki Sone

**LATVIA**

Dr. Ineta Vasaraudze

Dr. Juris Vitols

Dr. Inta Dinsberga

Mr. Antons Babuskins

**LITHUANIA**

Dr. Regina Giliaseviciene

Dr. Zaneta Kasilovskiene

Dr. Ieva Brimiene

**NORWAY**

Dr. Jenny Alvirovic

Dr. Theresia Kasin

Dr. Tonje Bohlin

**POLAND**

Prof. Tomasz Rechberger

Prof. Jan Kotarski

Dr. Dagmara Makowska-Mainka

Dr. Malgorzata Janik

Dr. Tomasz Kolossa

Dr. Janusz Tomaszewski

Dr. Krzysztof Wilk

Dr. Wojciech Majkusiak

Prof. Jacek Suzin

Prof. Rafal Kurzawa

Dr. Agnieszka Harabin-Matysiak

**SLOVAKIA**

Dr. Alexander Brestansky

Dr. Peter Krizo

Dr. Peter Salko

**SPAIN**

Dr. Josep Perello Capo

Dr. Gregorio López González

Dr. José Cruz Quílez Conde

Dr. Juan Gilabert Estelles

**UNITED STATES**

Dr. Anthony Adams

Dr. Janeen Arbuckle

Dr. Samuel Alexander

Dr. George Besong

Dr. David Archer

Dr. Sarah Artman

Dr. Soyini Hawkins

Dr. Andrea Galloway

Dr. Andrea Lukes

Dr. Robin Kroll

Dr. Franklin Morgan

Dr. Ratna Palakodeti

Dr. Sandra Hurtado

Dr. James H. Liu

Dr. Ozgul Muneyyirci-Delale

Dr. Brad Chesney

Dr. D. Harnsberger

Dr. Eric Brown

Dr. Stephen Pound

Dr. Issam Daya

Dr. Allan Dinnerstein

Dr. Ramin Farsad

Dr. Robert Parker

Dr. Michael Moore

Dr. Phillip Madonia

Dr. Yvette Poindexter

Dr. Elizabeth Puscheck

Dr. Alexandra Pellicena

Dr. James Simon

Dr. Robert Smith

Dr. Carrie Swartz

Dr. Gregory Swor

Dr. Stuart Weprin

Dr. Soumia Brakta

Dr. Ali Bajwa

Dr. Judith Jordan Williams

Dr. Kevin Fleishman

Dr. Ronnie Givens

Dr. Rowell Ashford II

Dr. Jay Staub

Dr. Craig Saffer

Dr. Botros Rizk

Dr. Jeffrey Baker

Dr. Valerie Sorkin-Wells

Dr. Edmond Pack

## Supplementary Methods

**Participating regions and countries**

The following regions and countries participated in SCHUMANN and enrolled participants: Europe (Austria, Belgium, Bulgaria, Czech Republic, Estonia, Finland, Germany, Greece, Hungary, Italy, Latvia, Lithuania, Norway, Poland, Slovakia, Spain); Canada; China; Japan; and the United States of America.

**Full inclusion criteria**

**Age**

1. Participants must be ≥18 years of age at the time of signing the informed consent form.

**Type of participant and disease characteristics**

1. Participants must have visually confirmed endometriosis: the detection of endometriotic lesions during laparoscopy or laparotomy (with or without pathologic diagnosis) within 10 years but not more recently than 8 weeks from visit 1a (surgically diagnosed endometriosis). In Japan only (and for no more than half of all randomized Japanese participants), the diagnosis can be based on previous imaging (i.e., an endometriosis lesion detected by ultrasound or magnetic resonance imaging [MRI]). If the participant has been diagnosed by ultrasound, the lesion must be visualized again using ultrasound at the screening visit. If the participant has been diagnosed using MRI, the diagnosis (clinically diagnosed endometriosis) must have been made within 12 months of visit 1a.
2. Both sub-criteria involving pain symptoms must be fulfilled:

- At visit 1a, the participant presents self-reported moderate-to-severe pain which—in the investigator’s judgment—is reasonably likely to translate into sufficiently severe pain symptoms to fulfill the eligibility criteria and be caused by endometriosis, and
- During the screening period, at least 24 daily endometriosis-symptom-diary (ESD) entries are made during 28 consecutive days, starting on the first day with menstrual bleeding at or after visit 1a; in addition, ESD entries marked item 1a (“worst pain”) on the daily numeric rating scale total 98 or more.

1. Willingness to use standardized rescue pain medications for endometriosis-associated pelvic pain (EAPP) (i.e., ibuprofen, acetaminophen, and tramadol) and not use any prophylactic pain medication, in accordance with the investigator’s instructions.
2. Ability to swallow the study intervention, i.e., different kinds of tablets, as complete units.
3. Good general health (except for findings related to endometriosis), confirmed by the subject’s medical history, physical and gynecologic examinations, and laboratory test results.
4. Normal or clinically insignificant cervical cytology not requiring further follow-up:
   - A cervical cytology sample must be obtained during screening, or
   - A documented normal result must be available from cervical cytology conducted within 12 months prior to visit 1a.
   - Human papilloma virus (HPV) testing in participants with atypical squamous cells of unknown significance (ASCUS) will be used automatically as an adjunctive test. Participants with ASCUS can be included if they are negative for high-risk HPV strains.

**Sex**

1. Female.

Contraceptive use by participants should be consistent with local regulations regarding methods of contraception for those participating in clinical studies.

Participants must be willing to use a non-hormonal barrier method of contraception (spermicide-coated condoms for their partners) between the screening visit and the end of the study. This is not required if adequate contraception can be achieved through vasectomy of the male partner(s), female sterilization, the use of a copper intrauterine device for at least 6 months, or total abstinence from heterosexual intercourse (for details refer to section 10.4).

**Informed consent**

1. Capable of providing signed informed consent, which includes compliance with the requirements and restrictions listed in the informed consent form and in this protocol.

**Full exclusion criteria**

**Medical conditions**

1. Current pregnancy or less than 3 months since delivery, abortion, or the end of lactation by visit 1a.
2. Hypersensitivity to any ingredient in the study intervention and/or the standardized rescue medications (see the latest available version of the investigators’ brochure for BAY1817080 and the placebo, as well as the prescriber information for elagolix and the standardized rescue medications).
3. Known osteoporosis.
4. History of low-trauma fracture.
5. Contraindications for elagolix or the standardized rescue medications.
6. Current malignancy or history of cancer (exception: basal cell or squamous cell carcinoma of the skin) within the 5 years prior to visit 1a.
7. Any other disease or condition that the investigator believes may compromise the function of body systems, potentially resulting in altered absorption, excessive accumulation, impaired metabolism, or altered excretion of the study intervention (e.g., chronic bowel diseases, Crohn’s disease and ulcerative colitis).
8. Menopause or signs of menopausal transition, including absence of regular menstrual cycles, based on investigator’s judgment (absence of information regarding menstrual bleeding pattern e.g., due to long term use of hormonal contraception, is not an exclusion criterion).
9. Any disease or condition that may worsen during the study period based on the investigator’s assessment and opinion.
10. Uterine bleeding with abnormal regularity or heaviness (excluding heavy menstrual bleeding that does not require treatment).
11. Any findings that require further diagnostic procedures to avoid harm to the participant (e.g., ovarian tumors of uncertain origin or pelvic masses of unclear etiology).
12. Any serious or unstable diseases or medical conditions, including psychiatric disorders, which might interfere with the conduct of the study or the interpretation of the result, including for example:

- a history of hysterectomy and/or bilateral oophorectomy
- any conditions that the investigator believes could contribute significantly to pelvic pain, e.g., fibromyalgia, uterine fibroids, irritable bowel syndrome, or other bowel disorders
- any other underlying diseases requiring the regular use of pain medication (e.g., migraine)
- a history of or current anxiety or depression, unless stable with or without medical treatment ≥6 months before visit 1a.

1. Major surgery scheduled during the study period.
2. The non-responsiveness of EAPP to earlier treatment with gonadotropin-releasing hormone (GnRH)-agonists or GnRH-antagonists, based on the investigator’s judgment.
3. Severe acute respiratory syndrome coronavirus 2 (SARS-CoV-2) positive virus ribonucleic acid (RNA) test within 4 weeks prior to visit 1a, as reported by the participant, regardless of symptoms.
4. A history of COVID-19 infection with persistent/ongoing symptoms.
5. Contact with a SARS-CoV-2- positive or COVID-19 patient within the 4 weeks prior to visit 1a.

**Prior/concomitant therapy**

1. Intake of medication prohibited due to potential drug–drug interaction (see Section •); local administration is permitted.
2. Use of other treatments that might interfere with the conduct of the study or the interpretation of the results, including:

- hormonal medications (see Section 6.8.2.2)
- other treatments intended for endometriosis/pelvic pain during study participation, including the use of herbal products or traditional Chinese medicine for symptom relief but excluding standard rescue pain medications.

**Prior/concurrent clinical-study experience**

1. Simultaneous participation in another clinical trial investigating medicinal product(s). Participation in another trial 3 months prior to visit 1a that might have an impact on the study objectives (at the discretion of the investigator).
2. Previous assignment to a study intervention (randomization) in the present study (re-including previously randomized participants in the study may lead to bias).

**Diagnostic assessments**

1. Laboratory values outside the inclusion range (specified in the laboratory manual) and considered clinically relevant.
2. Liver-safety-related exclusion criteria:
   - Alanine aminotransferase (ALT) above 2 × upper limit of normal (ULN), or
   - Aspartate aminotransferase (AST) above 2 × ULN, or
   - Total bilirubin greater than ULN, or
   - Alkaline phosphatase (AP) above 2 × ULN, or
   - An international normalized ratio greater than ULN, or
   - Positive hepatitis B virus surface antigen, or
   - Positive hepatitis C virus (HCV) antibodies and detected mRNA (HCV-mRNA tested for only if hepatitis C virus antibodies are detected).
3. Severe hypertension, defined as a sitting systolic blood pressure ≥160 mmHg and/or diastolic blood pressure ≥100 mmHg.
4. Estimated glomerular filtration rate (eGFR) <30 mL/min/1.73 m^2^, calculated using the Modification of Diet in Renal Disease formula. This study uses a different eGFR formula for participants enrolled at sites in Japan.

**Other exclusions**

1. Desire to become pregnant during the study period.
2. Close affiliation with the study site, e.g., a close relative of the investigator, dependent person (e.g., employee or student of the site, or sponsor’s staff).
3. Inability to cooperate with the study procedures for any reason, e.g., language comprehension or inability to access the study site.
4. Abuse of alcohol or medicines, or the use of recreational/illicit drugs, as evaluated by the investigator.
5. Otherwise vulnerable patients. Patients who are in custody by order of an authority or a court of law.

**Additional information on procedures**

**Electronic patient-reported outcomes (PROs)**

PROs were collected using an electronic handheld diary (eDiary), except for the pain catastrophizing score (PCS) and painDETECT, which were collected as paper questionnaires. Study participants were expected to respond to the PROs without any assistance. Both site personnel and study participants received validated training [1, 2] to ensure accurate and reliable reporting of pain scores and the proper use of pain scales, as well as to minimize the placebo response. Training was provided at the start of the study; retraining was provided at regular intervals and as needed during the course of the trial.
The following PROs were used:

1. **Endometriosis symptom diary (ESD)**

The ESD v 9.0 is a multi-item PRO instrument designed to assess the patient’s experience of endometriosis symptoms. Item 1 in the ESD served as the primary endpoint in this study. This PRO was first used in a validation study [3] and subsequently in a treatment study with an aromatase inhibitor [4]. Regarding the rationale for developing the ESD, see Gerlinger et al. 2012 [5]. To evaluate psychometric properties, ESD pain items were complemented by pain recordings using a 4-week recall visual-analog scale.

The study participant was expected to complete the ESD every evening (between 18:00 and 24:00, preferably after the evening dose), using the diary entry to reflect on her experiences over the previous 24 hours. Participants were asked to record their:

- endometriosis pain at its worst (single items 1–3)
- vaginal bleeding (single item 4)
- sexual intercourse and dyspareunia (single items 5–7)
- use of the study intervention (single item 8)
- use of standardized rescue medication and other pain medication (single items 9–11).

1. **Endometriosis impact scale (EIS)**

The EIS version 5.0 is a multi-item PRO instrument designed to assess the impact of endometriosis symptoms on patients’ lives. The EIS was used in a validation study [3] and subsequently in a treatment study with an aromatase inhibitor [6]{, #310;, #310}. The rationale for developing the EIS is documented in Gerlinger et al. 2012 [5].

The study participant was expected to complete the EIS once a week (between 18:00 and 24:00), reflecting on her experiences during the previous 7 days and recording the effect of endometriosis pain on her daily life by describing its impact on:

- physical activities (physical subscale; contains 7 single items)
- emotional effect (emotional subscale; contains 7 single items)
- sexual activities (sexual activities subscale; contains 3 single items)
- ability to concentrate
- ability to sleep
- household activities
- paid work or study
- social and leisure activities

1. **Visual analog scale (VAS) for pelvic pain**

The VAS is commonly used to assess pain in various indications. It has been used to develop Visanne^®^ in endometriosis, allowing for comparison with historical data [7]. The VAS consists of a straight line with 101 positions/dots to choose from and verbal anchors at either end, representing a continuum of pain intensity. One end of the line has the anchor “no pain” while the other end of the line has the anchor “unbearable pain.” Study participants were asked to indicate their level of pelvic pain (i.e., endometriosis pain in the lower abdomen and surrounding areas) over the past 4 weeks by making a single vertical mark on the line. The VAS had to be completed in the eDiary during site visits.

1. **Patient global impression of severity (PGI-S) and change (PGI-C)**

PGI-S and PGI-C are self-administered instruments used in previous endometriosis trials. They were included in this study to investigate the psychometric properties of the ESD and EIS. PGI-S asks the participant to rate the severity of her endometriosis pain over the past 4 weeks using a 6-point verbal single-item rating Likert scale (no pain, very mild, mild, moderate, severe, and very severe). The PGI-C measures record changes in the participant’s endometriosis pain since the start of the study intervention, using a single-item 5-point Likert scale, with responses ranging from “much better” to “much worse.” Participants had to complete both PGI-S and PGI-C in their eDiaries during site visits.

1. **PainDETECT**

The painDETECT PRO was developed and its properties were tested on a sample of lower-back pain patients; Freynhagen et al. [8] have summarized additional PRO and clinical research. There is limited evidence for the applicability and validity of painDETECT in endometriosis; it was used, with data intended for evaluation, as an explorative endpoint. PainDETECT data were collected via a paper questionnaire to explore and describe the potential presence of neuropathic pain and the change under intervention. The painDETECT is a self-administered 13-item paper questionnaire designed to screen patients with neuropathic pain [8]. It includes 3 domains:

- - pain-course pattern
  - pain radiation
  - sensory symptoms.

A painDETECT score is generated for each participant by assigning a value to items 7–13 (ranging from 0 = “never” to 5 = “very strongly”) and determining the total. This score is then adjusted, based on responses to items about pain-course pattern and pain radiation. The final score ranges from -1 to 38. For screening purposes, the below cut-off points have been suggested in other indications and were used in this study exploratively, as there is limited evidence of using painDETECT and the validity of these thresholds for endometriosis indication:

- score ≤12, a neuropathic component is unlikely (<15%)
- score ≥19, a neuropathic component is likely (>90%)
- Between these scores, the result is uncertain, i.e., a neuropathic pain component may be present.

1. **EuroQoL 5-dimension 5-level (EQ-5D-5L)**

The EQ-5D-5L is a self-administered preference-based generic measure of health status, which includes five dimensions: mobility, self-care, usual activities, pain/discomfort, and anxiety/depression [9]. Patients rate each question using the following five-point Likert scale: having no problems, having slight problems, having moderate problems, having severe problems, and being unable to cope or having extreme problems. In addition, patients are asked to self-rate their own health today on a vertical 0–100-unit visual analog scale (VAS), with 0 corresponding to "the worst health you can imagine,” and 100 corresponding to "the best health you can imagine.” The EQ-5D-5L was collected via the eDiary and programmed after weekly EIS data collection during the screening phase before treatment and monthly (every 4 weeks) during the treatment phase and follow-up.

1. **Modified work productivity and activity-impairment questionnaire (mWPAI)**

The Work Productivity and Activity Impairment Questionnaire is commonly used to assess the impact of different health conditions, including gynecologic indications in relation to absenteeism, presenteeism, and activity impairment [10]. A modified version for Endometriosis (mWPAI) with 6 items involving recall over the previous 4 weeks was used; data were collected via the eDiary during site visits, as indicated in the SoA [11, 12].

1. **Pain catastrophizing scale (PCS)**

The pain catastrophizing scale (PCS) is a 13-item instrument scale derived from catastrophizing concepts described in the literature [13, 14] and items from the catastrophizing subscale of the Coping Strategy Questionnaire [15].

The PCS instructions ask patients to reflect on past painful experiences and to indicate the degree to which they experienced each of 13 thoughts or feelings when experiencing pain; they indicate their responses using 5-point scales, with responses ranging from (0) not at all to (4) all the time. The PCS yields a total score and three subscale scores that assess rumination, magnification, and helplessness. The final score is computed by totaling responses to all 13 items. PCS total scores range from 0 to 52 [16]. PCS data were collected on paper at baseline as an explorative endpoint to better understand the pain-related distress experienced by women with endometriosis.

1. **Additional daily symptom items**

Three additional items were asked for and reported daily in the eDiary to assess dyschezia (pain associated with bowel movement), dysuria (pain associated with urination), and the number of hot flushes (the sensation of heat with sweating). These questions were completed by the study participant each evening (between 18:00 and 24:00) alongside the ESD. Participants were asked to report pain at its worst during the previous 24 hours on a 0–10 NRS scale in order to assess dyschezia and dysuria. These items were derived from previous qualitative research and ESD development. In addition, the number of hot flushes during the previous 24 hours was reported to assess the tolerability of various active treatments.

**Additional information on safety-related assessments**

In addition to recording adverse events throughout the study, the following safety assessments were performed:

- The investigator carried out a comprehensive physical examination at screening and follow-up.
- A gynecologic examination including a transvaginal ultrasound was performed by the investigator at screening, baseline, Day 84, and follow-up. Cervical cytology was conducted at screening.
- Vital signs (blood pressure and heart rate) were assessed at screening and every second site visit.
- A urine pregnancy test was performed at screening, baseline, every 4 weeks during treatment, and during the follow-up period.
- Samples for safety laboratory assessments were taken at screening, every 4 weeks during treatment, and during the follow-up period. Those included:
  - Biochemistry: Albumin, Calcium, Magnesium, Creatinine, Cystatin C, Phosphorus, Potassium, Sodium, Total protein, Blood urea nitrogen, Alkaline phosphatase, Lipase, Pancreatic α-amylase, Non-fasting glucose, Total cholesterol, high-density lipoprotein cholesterol, low-density lipoprotein cholesterol, Triglycerides, β-human chorionic gonadotropin and Ferritin.
  - Hematology: Erythrocytes, Hematocrit, Hemoglobin, Leukocytes with differentials, Platelets, and Hemoglobin A1c.
  - Coagulation: Activated Partial Thromboplastin Clotting Time, Fibrinogen, International Normalized Ratio, Prothrombin Time (Quick), Antithrombin III.
  - Hormones: Estradiol and Progesterone.
- An increase in liver function parameters was regarded as a potential risk at the beginning of the phase 2 program with eliapixant, based on animal data (data not shown). Therefore, blood samples used for testing liver parameters, i.e., AP, ALT (glutamic-pyruvic transaminase), AST (glutamic oxaloacetic transaminase), Gamma-glutamyl transferase, and total bilirubin, were taken every 2 weeks from all patients randomized to eliapixant or placebo (every 4 weeks from patients on elagolix). The safety-related liver monitoring was accompanied by rules for initiating close liver observation and defining discontinuation criteria. A close observation in line with US FDA guidelines for assessing potential drug-induced liver injury [17] had to be initiated during the intervention or follow-up phase if ALT or AST reached >3 × ULN. Close observation included:
  - Sampling for the first batch of laboratory parameters (see Table S2).
  - Repeating follow-up samplings (see Table S2) 2 to 3 times per week. Frequency of retesting can decrease to once a week or less if abnormalities stabilize.
  - Obtaining a detailed history of the symptoms and prior or concurrent diseases.
  - Obtaining a history of concomitant drug use (including nonprescription medications and herbal and dietary supplement preparations), alcohol use, recreational drug use, and special diets.
  - Ruling out acute viral hepatitis, autoimmune or alcoholic hepatitis, nonalcoholic steatohepatitis, hypoxic/ischemic hepatology, and biliary tract disease. This might require performing additional procedures, e.g., ultrasound examinations. If requested, tests could be performed retrospectively using residual blood/serum samples collected at visits before the laboratory abnormalities occurred.
  - Obtaining a history of exposure to environmental chemical agents.
  - Obtaining additional tests to evaluate liver function as required (e.g., INR, direct bilirubin measurements).
  - Liver imaging.
  - Considering gastroenterology or hepatology consultations.

**Prohibited medications**

**Pain medication other than permitted standardized rescue medication**

- Use of all other analgesics beyond the permitted rescue pain medication was discouraged during the study on grounds that it could potentially disturb the study outcome.
- If any other pain medication was taken, for any reason (including EAPP), it had to be recorded in the electronic case report form.

**Hormonal medications**

- Hormonal medications listed in Table S3 were prohibited during participation in this study.
- Participants using hormonal treatment at visit 1a had to be willing to stop this at the next suitable time point at or following visit 1a, as outlined in Table S3.

**Medication prohibited due to potential drug–drug interaction**

**Strong CYP3A4 inducers and strong CYP3A4 inhibitors**

The following strong CYP3A4 inducers and inhibitors were prohibited within the 4 weeks prior to the start of the study intervention and during the intervention period of the study:

- **CYP3A4 inducers:** e.g., St. John's wort, rifampicin, carbamazepine, phenytoin
- **CYP3A4 inhibitors**: e.g., itraconazole, posaconazole, voriconazole, ritonavir, nelfinavir, or other inhibitors of human immunodeficiency virus protease, clarithromycin, telithromycin, nefazodone, telaprevir, boceprevir

Note: ketoconazole and other triazole antifungal drugs were allowed for topical/local use (including vaginal application).

**Strong OATP1B1 inhibitors**

- Strong organic anion transporting polypeptide (OATP) 1B1 inhibitors (e.g., cyclosporine and gemfibrozil) were prohibited during the intervention period of this study.

**OATP1B1/1B3, BCRP, and P-gp substrates**

Based on preclinical data, an increase in exposure of OATP1B1/1B3, P-gp, and BCRP substrates during the co-administration of BAY 1817080, due to inhibition of those transporters by BAY 1817080, cannot be excluded.

- Apixaban, dabigatran, edoxaban, digoxin: prohibited from 2 weeks prior to start of treatment until 2 weeks after the end of the intervention period in this study.
- Participants receiving a combination of BAY 1817080 and OATP1B1/1B3, BCRP, and P-gp substrates should be closely monitored for signs and symptoms of adverse events, due to their increased exposure to co-administered OATP1B1/1B3, BCRP, and P-gp substrates.
- Dose modification of OATP1B1/1B3, BCRP, and P-gp substrates must be considered based on the prescriber information or such compounds must be avoided. Typical OATP1B1, OATP1B3, BCRP, and P-gp substrates include but are not limited to the following: fexofenadine, sulfasalazine, rosuvastatin, atorvastatin, cerivastatin, glyburide, pravastatin, repaglinide, simvastatin.

**Additional information on the statistical analysis**

**Sample-size calculations**

The sample-size calculation assumed a true change in mean worst EAPP, as measured on the 11-point NRS by ESD item 1, after 12 weeks of placebo treatment of -2.3, a maximum mean worst EAPP change of -1.2 for eliapixant vs. placebo, a common standard deviation of 2.5 (derived from effects observed in previous studies conducted by the Sponsor in this indication), and a set of dose–response shapes, including two asymptotic maximum changes from the placebo effect (Emax) and two sigmoidal Emax models (Supplemental Fig. 2 and Supplemental Table 4).

**Primary endpoint analysis**

The multiple comparison procedure-modeling (MCP-Mod) method [15], which combines multiple comparison procedure principles with modeling techniques, was used to carry out the primary analysis of the primary efficacy endpoint. This method allows modeling flexibility for dose estimation, while preserving the robustness needed to model misspecifications associated with MCP procedures. More specifically, this study uses a generalized version of the original MCP-Mod method, which allows dose–response testing and modeling in conjunction with the response variable described by a parametric model [16]. The generalized MCP-Mod approach (i.e., adjusted for baseline mean worst EAPP [to account for potential baseline imbalances] and geographic region [for administrative purposes and to account for ethnic and etiologic differences]) takes multiplicity into account [15, 16].

For MCP-Mod, the four candidate models were as follows: an Emax model with parameters ED_50_=25, an Emax model with parameters ED_50_=100, a sigmoidal model with parameters ED_50_=35 and Hill coefficient=3, and a sigmoidal model with parameters ED_50_=75 and Hill coefficient=5. All of these candidate models assumed a monotonically decreasing dose response. All model parameters shown in Supplemental Table 4 were based on phase 2a eliapixant data in patients with RCC [18] and past studies carried out by the Sponsor in patients with endometriosis . The dose–response relationships of the candidate models are shown in Supplemental Fig. 2.

The estimand of interest, needed to assess the dose–response relationship for the primary efficacy endpoint, was the effect of the received intervention in women with surgically confirmed symptomatic endometriosis and no validity findings affecting efficacy. The attributes of the estimand were as follows:

Population: women with surgically confirmed symptomatic endometriosis, as further defined by the inclusion/exclusion criteria described in the study protocol.

Variable: absolute change in mean worst EAPP from baseline (within 28 days of the first intake of study drug) to the end of the intervention (last 28 days, ending with the last intake of study drug planned on Day 84 [+3]).

Treatment: eliapixant 25 mg or 75 mg or 150 mg or a placebo, plus standardized rescue medication taken for any reason.

Population-level summary: estimated model-based group mean of the primary endpoint’s values, adjusted for region and baseline EAPP. The treatment effect is evaluated as a difference between group means.

Intercurrent events: the premature discontinuation of the study intervention (due to a non-COVID-19-related AE, lack of efficacy, or any other reason); non-compliance with the study intervention; or an increased or decreased intake of the standardized rescue medication.

The intercurrent events that led to premature discontinuation of study intervention, due to a non-COVID-19-related AE or lack of efficacy, were interpreted as treatment failure and addressed by the composite strategy. A premature discontinuation of the study intervention for any other reason was addressed using a hypothetical strategy within the assumed hypothetical scenario that participants would not have left the study and would have had outcomes similar to those of other study participants. Intercurrent events involving non-compliance or a change in rescue medication were addressed by the treatment policy strategy.

**Definition of analysis sets**

**Full-analysis set**

All participants randomly assigned to the study intervention. Participants were analyzed based on the intervention they were randomized to.

**Safety-analysis set**

All participants randomly assigned to the study intervention who took at least 1 tablet as part of the study intervention. Participants were analyzed based on the intervention they actually received.

**Primary full-analysis set**

Subset of full-analysis set, containing all participants outside China with surgically confirmed endometriosis, randomly assigned to the study intervention. Participants were analyzed based on the intervention they were randomized to.

**Per-protocol set**

All participants randomly assigned to the study intervention, who took at least 1 tablet as part of the study intervention and had no validity findings affecting efficacy.

A list of potential validity findings was provided in a separate document listing important deviations and validity specifications; this was finalized before the unblinding. Participants were assigned to this analysis set based on the assessment meetings.

Participants missing baseline ESD item 1 and those without a valid post-baseline 28-day average of ESD item 1 were excluded from the per-protocol set.

Participants were analyzed based on the intervention they actually received.

**Primary per-protocol set**

Subset of the per-protocol set containing all participants outside China with surgically confirmed endometriosis. The analyses performed on the primary per-protocol set were chosen as the primary analyses.

**Use of analysis sets**

All efficacy endpoints were analyzed using the (primary) per-protocol set. Sensitivity analyses of the primary and secondary endpoints were performed using the (primary) full-analysis set. Safety endpoints were analyzed within the safety-analysis set.

## Supplementary results

**Other endpoints**

The EAPP scores and their changes from baseline after 4 and 8 weeks are shown in Supplementary Table 1. After 4 weeks, EAPP reductions were observed in all treatment groups, ranging from –0.83 (0.97) in the eliapixant 25 mg group to –1.49 (1.51) in the elagolix 150 mg group. These reductions further increased at Week 8, ranging from –1.26 (1.19) in the eliapixant 25 mg group to –2.78 (2.11) in the elagolix 150 mg group.

Rescue-medication use at baseline varied considerably between the treatment groups. Rescue-medication use was lowest in the eliapixant 75 mg arm, with a mean (SD) overall number of tablets (ibuprofen 400 mg, acetaminophen/paracetamol 500 mg, tramadol 50 mg) of 19.2 taken during the 28-day baseline period. Similar levels of overall rescue-medication use were observed in the eliapixant 25 mg and 150 mg groups and in the placebo group, with quantities ranging from 30.8 to 37.5 tablets. Higher levels of rescue-medication use were observed in the elagolix 150 mg arm with a mean of 50.9 tablets at baseline. However, levels of rescue-medication use ranged widely in all treatment arms, from no use at all in all treatment arms, to more than 140 tablets in all but the eliapixant 75 mg arm. These differences in means and ranges can also be observed when considering the three types of rescue medication separately. A large difference was observed in the use of opioids (i.e., tramadol 50 mg). The baseline mean number of tablets in the elagolix 150 mg group was 4.9—considerably higher than the mean number of tablets in the other groups (0.4 to 3.0). After 12 weeks of treatment, rescue-medication use was considerably lower in all treatment groups (–14.0 tablets across all treatment groups), with stronger reductions observed in treatment groups with higher baseline values. The impact of the various interventions on rescue medication is difficult to assess, due to large baseline differences.

Assessments based on the EIS showed some baseline differences between treatment groups, with both physical-activity and emotional-wellbeing scores being lower (indicating a better quality of life) in the eliapixant 25 mg, 150 mg, and placebo groups (transformed score means of 33.9 to 39.1 for the physical activity score and 36.7 to 38.9 for the emotional wellbeing score) than in the eliapixant 150 mg and elagolix 150 mg groups (means of 47.8 and 44.7 for physical activity and 43.0 in both groups for emotional wellbeing). After 12 weeks of intervention, EIS-assessed quality of life improved in all treatment groups. In the physical-activity domain, the reduction in scores ranged from –11.1 in the eliapixant 25 mg group to –25.5 in the elagolix 150 mg group. In the emotional wellbeing domain, the reduction in scores ranged from –11.7 in the placebo group to –18.6 in the elagolix 150 mg group. These improvements were in line with the changes observed on EAPP, with largest improvements consistently observed in the elagolix 150 mg group and differences between placebo and the different doses of eliapixant being small and clinically irrelevant.

PainDETECT mean scores were similar between treatment groups at baseline, ranging from 16.3 to 17.3. At end of treatment, reductions in PainDETECT scores were observed in all treatment groups, with means between –2.6 and –3.9 across the eliapixant and placebo arms, and a mean reduction of –6.7 in the elagolix 150 mg arm. Consistent with other endpoints, there were no relevant differences between the different doses of eliapixant and the placebo.

## Supplementary tables and figures


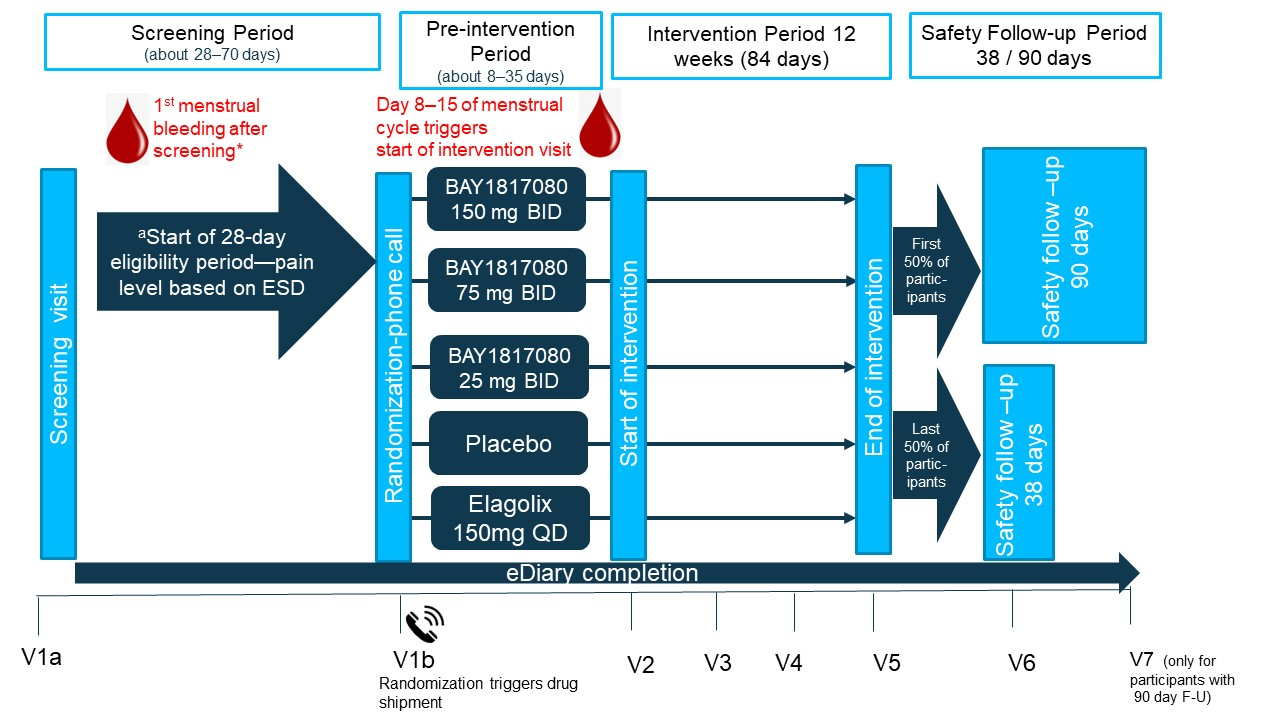


Fig. S1 SCHUMANN study design.
^a^Follow-up 2 was performed on all randomized participants, apart from those randomized to the open-label elagolix arm. Follow-up 2 was extended to 90±3 days after the last administration of the study intervention. In addition to the visits displayed in the schema, 5 additional visits (2a, 3a, 4a, 5a, and 6a) were added to take blood samples for liver monitoring.
The additional visits and the requirement to have follow-up 2 performed were implemented as part of a comprehensive safety follow-up in the context of the early termination of the study.

*Abbreviations:* *BID* twice daily, *ESD* endometriosis symptom diary


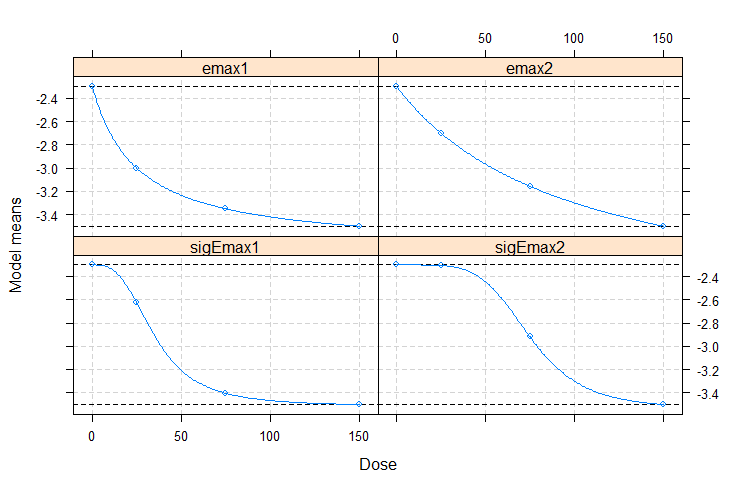


**Fig. S2** Candidate set of dose-response curves.

*Abbreviations:* *E_max_* maximum effect attributable to the intervention

**Supplementary Table 1** Mean worst EAPP and absolute change from baseline to Weeks 4 and 8 (full-analysis set)

| **Timepoint** | | **Eliapixant 25 mg BID** | **Eliapixant  75 mg BID** | **Eliapixant  150 mg BID** | **Placebo** | **Elagolix  150 mg** | **Total** |
| --- | --- | --- | --- | --- | --- | --- | --- |
| **Baseline** | n | 39 | 38 | 38 | 37 | 38 | 190 |
|  | Missing | 5 | 6 | 5 | 6 | 3 | 25 |
|  | Mean (SD) | 6.11 (1.70) | 6.11 (1.79) | 6.68 (1.60) | 5.92 (1.92) | 6.38 (1.76) | 6.24 (1.75) |
|  | Median | 6.30 | 6.05 | 6.88 | 5.65 | 6.19 | 6.35 |
|  | Q1, Q3 | 4.61, 7.36 | 4.86, 7.46 | 5.96, 7.61 | 4.56, 7.00 | 5.04, 8.17 | 4.89, 7.54 |
|  | Min, Max | 3.1, 9.6 | 3.0, 9.6 | 3.0, 9.9 | 2.6, 10.0 | 3.2, 9.3 | 2.6, 10.0 |
| **Week 4** | n | 37 | 33 | 35 | 35 | 35 | 175 |
|  | Missing | 7 | 11 | 8 | 8 | 6 | 40 |
|  | Mean (SD) | 5.30 (1.72) | 5.12 (2.12) | 5.65 (1.85) | 4.82 (1.82) | 4.83 (2.29) | 5.14 (1.97) |
|  | Median | 5.56 | 5.36 | 5.30 | 4.70 | 4.68 | 5.16 |
|  | Q1, Q3 | 3.93, 6.50 | 3.55, 6.52 | 4.52, 6.88 | 3.36, 6.36 | 2.57, 6.78 | 3.55, 6.61 |
|  | Min, Max | 2.6, 8.3 | 0.8, 9.4 | 2.3, 9.7 | 1.8, 8.5 | 0.3, 9.3 | 0.3, 9.7 |
| **Change from Baseline to Week 4** | n | 37 | 33 | 35 | 35 | 35 | 175 |
|  | Missing | 7 | 11 | 8 | 8 | 6 | 40 |
|  | Mean (SD) | –0.83 (0.97) | –0.91 (2.00) | –1.02 (1.54) | –1.15 (1.17) | –1.49 (1.51) | –1.08 (1.47) |
|  | Median | –0.69 | –0.49 | –0.80 | –0.99 | –1.11 | –0.86 |
|  | Q1, Q3 | –1.38, -0.19 | –1.50, -0.05 | –1.50, -0.06 | –1.52, -0.51 | –1.96, -0.67 | –1.66, -0.19 |
|  | Min, Max | –3.5, 0.6 | –8.2, 2.4 | –6.0, 1.5 | –4.2, 1.0 | –6.3, 1.2 | –8.2, 2.4 |
| **Week 8** | n | 33 | 28 | 31 | 32 | 28 | 152 |
|  | Missing | 11 | 16 | 12 | 11 | 13 | 63 |
|  | Mean (SD) | 4.68 (1.72) | 4.42 (2.50) | 4.92 (2.34) | 4.35 (1.80) | 3.41 (2.23) | 4.38 (2.16) |
|  | Median | 4.39 | 4.32 | 4.64 | 4.21 | 3.46 | 4.29 |
|  | Q1, Q3 | 3.38, 5.84 | 3.00, 6.16 | 3.63, 6.48 | 2.98, 5.67 | 1.92, 4.83 | 3.04, 5.79 |
|  | Min, Max | 1.7, 8.1 | 0.0, 9.4 | 0.0, 9.8 | 1.4, 8.1 | 0.0, 7.9 | 0.0, 9.8 |
| **Change from Baseline to Week 8** | n | 33 | 28 | 31 | 32 | 28 | 152 |
|  | Missing | 11 | 16 | 12 | 11 | 13 | 63 |
|  | Mean (SD) | –1.26 (1.19) | –1.87 (2.39) | –1.67 (2.30) | –1.66 (1.49) | –2.78 (2.11) | –1.82 (1.97) |
|  | Median | –0.96 | –1.02 | –1.09 | –1.40 | –2.38 | –1.32 |
|  | Q1, Q3 | –2.24,  –0.32 | –2.63,  –0.21 | –2.91,  –0.11 | –2.61,  –0.63 | –3.73,  –1.13 | –2.71,  –0.39 |
|  | Min, Max | –4.1, 0.6 | –9.0, 1.0 | –8.3, 1.6 | –5.4, 1.2 | –7.8, 0.3 | –9.0, 1.6 |

*Abbreviations: BID* twice daily*, EAPP* endometriosis-associated pelvic pain (item 1 in the endometriosis symptom diary [*ESD*]), *Q1* first quartile, *Q3* third quartile, *SD* standard deviation

Supplemental Table S2 Samples taken under close observation to ensure liver safety

| **Parameters** | **Time point(s)** | **Comment** |
| --- | --- | --- |
| **First batch of parameters to be taken under close observation to ensure liver safety** | | |
| Albumin | As needed | First sample after AST or ALT >3 × ULN |
| Alkaline phosphatase (AP) |  |  |
| Alanine-aminotransferase (ALT) |  |  |
| Aspartate-aminotransferase (AST) |  |  |
| Complete blood count including WBC with differentials |  |  |
| Cholinesterase |  |  |
| Conjugated (direct) bilirubin |  |  |
| Creatine kinase (CK) |  |  |
| GGT |  |  |
| Hemoglobin |  |  |
| INR |  |  |
| Lactate dehydrogenase (LDH) |  |  |
| Prothrombin time |  |  |
| Total bilirubin |  |  |
| HDL-cholesterol |  |  |
| LDL-cholesterol |  |  |
| Total cholesterol |  |  |
| Triglycerides |  |  |
| Anti-hepatitis A virus IgM antibodies. |  |  |
| Hepatitis B virus surface antigen (if positive, automatically test below antibodies related to HBV and HDV)   - Anti-hepatitis B surface antibodies - Anti-hepatitis B core total antibodies - Anti-hepatitis B IgM antibodies - Hepatitis B PCR (viral copies) - Hepatitis D virus antibodies (if positive, automatically test HDV RNA) |  |  |
| Anti-hepatitis C virus antibodies (if positive, automatically test hepatitis C viral copies) HCV RNA |  |  |
| Anti-hepatitis D virus antibodies (if positive, automatically test HDV RNA) |  |  |
| Anti-hepatitis E virus IgM (if positive, automatically test HEV RNA) |  |  |
| Anti-cytomegalovirus (CMV) IgM antibodies |  |  |
| Anti-Epstein-Barr virus (EBV) IgM antibodies |  |  |
| Herpes simplex IgM (anti-HSV IgM) |  |  |
| IgG level (gamma globulins) |  |  |
| IgA |  |  |
| IgM |  |  |
| c-Antineutrophil cytoplasmic antibodies |  |  |
| c-Antineutrophil perinuclear antibodies |  |  |
| Anti-mitochondrial antibodies |  |  |
| Anti-nuclear antibodies (ANA) |  |  |
| Anti-smooth muscle antibodies (ASMA) |  |  |
| A1AT level |  |  |
| Ceruloplasmin |  |  |
| Ferritin |  |  |
| Iron |  |  |
| Total iron binding capacity |  |  |
| **Follow-up samples taken under close observation for liver safety** | | |
| Albumin | As needed | Additional parameters/tests may be added as medically justified |
| Alkaline phosphatase (AP) |  |  |
| Alanine-aminotransferase (ALT) |  |  |
| Aspartate-aminotransferase (AST) |  |  |
| WBC with differentials |  |  |
| Cholinesterase |  |  |
| CK |  |  |
| Conjugated (direct) bilirubin |  |  |
| Total bilirubin |  |  |
| GGT |  |  |
| INR |  |  |
| LDH |  |  |
| *Abbreviations:* *A1AT* alpha-1 antitrypsinm, *CK* creatine kinase, *DNA* deoxyribonucleic acid, *GGT* gamma-glutamyl transferase, *HBV* hepatitis B virus, *HDL* high-density lipoprotein, *HDV* hepatitis D virus, *HEV* hepatitis E virus, *HSV* herpes simplex virus, *IgA* immunoglobulin A, *IgG* immunoglobulin G, *IgM* immunoglobulin M, *INR* international normalized ratio, *LDH* lactate dehydrogenase, *LDL* low-density lipoprotein, *PCR* polymerase chain reaction, *RNA* ribonucleic acid, *ULN* upper limit of normal, *WBC* white blood cells | | |

Supplemental Table S3 Discontinuation of hormonal medications

| **Hormonal medications before the study** | **When hormonal medications were stopped** | **Bleeding that counts as the 1^st^ menstrual bleeding after visit 1a** |
| --- | --- | --- |
|  |  |  |
| Cyclic combined contraceptives (oral, vaginal, or transdermal) | Completed the current cycle ongoing at visit 1a | Withdrawal bleeding that occurs at the end of the cycle ongoing at visit 1a |
| Short-acting progestins or extended regimen of COC | Stopped at visit 1a | Withdrawal bleeding after stopping treatment |
| Hormone-releasing IUDs (Jaydess™, Kyleena™, Liletta™, and Mirena™) or subdermal implants | Not allowed during the study, users were only eligible if their IUD/implants were coincidentally removed shortly before visit 1a, normal menses resumed before visit 1a, and the participant was willing to wait to restart IUD/implant use until after the conclusion of the study. Removal of the IUD/implant for study participation only is not allowed | First menstrual bleeding after visit 1a (in participants entering the study without hormonal treatment) |
| GnRH-agonists or long-acting hormonal contraceptive (injectables) | Not allowed during the study; users are eligible only if the last injection was administered more than one application interval before visit 1a and normal menses have resumed before visit 1a | First menstrual bleeding after visit 1a (in participants entering the study without hormonal treatment) |
| GnRH-antagonists and progesterone receptor modulators | Not allowed during the study, users are only eligible if these have been stopped at least 28 days before visit 1a and normal menses have resumed before visit 1a | First menstrual bleeding after visit 1a (in participants entering the study without hormonal treatment) |
| Hormonal replacement treatments (e.g., add-back therapy for menopausal symptoms from GnRH-analogs) | Not allowed during the study, users are only eligible if these have stopped before visit 1a and normal menses have resumed before visit 1a | First menstrual bleeding after visit 1a (in participants entering the study without hormonal treatment) |

*Abbreviations: COC* combined oral contraceptive, *IUD* intrauterine device, *GnRH-a* gonadotropin-releasing hormone agonists

| Supplemental Table S4 Parameters of the dose-response curves in the candidate set | | | | |
| --- | --- | --- | --- | --- |
| Model | | Parameters | | |
| **Abbreviation** | **Indicator**$\boldsymbol{m}$ | $\boldsymbol{ED}_{\mathbf{50}}$ | $\boldsymbol{E}_{\boldsymbol{max}}$ | **Hill factor** $\boldsymbol{\eta}$ |
| emax1 | 1 | 25 | –1.4 | 1 |
| emax2 | 2 | 100 | –2.0 | 1 |
| sigEmax1 | 3 | 35 | –1.2 | 3 |
| sigEmax2 | 4 | 75 | –1.2 | 5 |

*Abbreviations: ED_50_* median effective dose, *E_max_* maximum effective dose

## Supplementary references

1. Treister R, Eaton TA, Trudeau JJ, Elder H, Katz NP. Development and preliminary validation of the focused analgesia selection test to identify accurate pain reporters. J Pain Res. 2017;10:319–26.

2. Treister R, Lawal OD, Shecter JD, Khurana N, Bothmer J, Field M, et al. Accurate pain reporting training diminishes the placebo response: results from a randomised, double-blind, crossover trial. PLoS One. 2018;13(5):e0197844.

3. ClinicalTrials.gov. BA6273B. Validation Study for Endometriosis (VALEPRO). 2013. Available from: https://www.clinicaltrials.gov/ct2/show/NCT01643122.

4. Mannucci PM. Hemostatic drugs. N Engl J Med. 1998;339(4):245–53.

5. Gerlinger C, Schumacher U, Wentzeck R, Uhl-Hochgräber K, Solomayer EF, Schmitz H, et al. How can we measure endometriosis-associated pelvic pain? Journal of Endometriosis. 2012;4(3):109–16.

6. ClinicalTrials.gov. PH-39016. 15832 (ESPARIOS) Bay98-7196, Dose Finding/POC Study. 2017.: Available from: https://clinicaltrials.gov/ct2/show/NCT02203331.

7. Gerlinger C, Schumacher U, Faustmann T, Colligs A, Schmitz H, Seitz C. Defining a minimal clinically important difference for endometriosis-associated pelvic pain measured on a visual analog scale: analyses of two placebo-controlled, randomized trials. Health Qual Life Outcomes. 2010;8:138.

8. Freynhagen R, Baron R, Gockel U, Tölle TR. painDETECT: a new screening questionnaire to identify neuropathic components in patients with back pain. Curr Med Res Opin. 2006;22(10):1911–20.

9. EuroQoL Group. EQ-5D-5L. 2017. Available from: https://euroqol.org/eq-5d-instruments/eq-5d-5l-about/ Version 18Apr2017.

10. Reilly MC, Zbrozek AS, Dukes EM. The validity and reproducibility of a work productivity and activity impairment instrument. Pharmacoeconomics. 1993;4(5):353–65.

11. Chaves JF, Brown JM. Spontaneous cognitive strategies for the control of clinical pain and stress. J Behav Med. 1987;10(3):263–76.

12. Spanos NP, Radtke-Bodorik HL, Ferguson JD, Jones B. The effects of hypnotic susceptibility, suggestions for analgesia, and the utilization of cognitive strategies on the reduction of pain. J Abnorm Psychol. 1979;88(3):282–92.

13. Rosenstiel AK, Keefe FJ. The use of coping strategies in chronic low back pain patients: relationship to patient characteristics and current adjustment. Pain. 1983;17(1):33–44.

14. Sullivan M. The pain catastrophizing scale. 1995. Available from: https://sullivan-painresearch.mcgill.ca/pdf/pcs/PCSManual_English.pdf.

15. European Medicines Agency (EMA). Qualification of MCP Mod as an efficient statistical methodology for model-based design and analysis of Phase II dose finding studies under model uncertainty. 2014. Available from: https://www.ema.europa.eu/en/documents/regulatory-procedural-guideline/qualification-opinion-mcp-mod-efficient-statistical-methodology-model-based-design-analysis-phase-ii_en.pdf. Accessed June 14, 2022.

16. Pinheiro J, Bornkamp B, Glimm E, Bretz F. Model-based dose finding under model uncertainty using general parametric models. Stat Med. 2014;33(10):1646–61.

17. US Food and Drug Administration. Drug-induced liver injury: premarketing clinical evaluation. 2009. Available from: https://www.fda.gov/regulatory-information/search-fda-guidancedocuments/drug-induced-liver-injury-premarketing-clinical-evaluation.

18. Morice A, Smith JA, McGarvey L, Birring SS, Parker SM, Turner A, et al. Eliapixant (BAY 1817080), a P2X3 receptor antagonist, in refractory chronic cough: a randomised, placebo-controlled, crossover phase 2a study. Eur Respir J. 2021;58(5):2004240.
